# Understanding COVID-19 vaccine hesitancy: A cross-sectional study in Malang District, Indonesia

**DOI:** 10.3389/fpubh.2022.1030695

**Published:** 2023-01-26

**Authors:** Sujarwoto, Asri Maharani, Holipah, Sri Andarini, Rindi Ardika Melsalasa Saputri, Eduwin Pakpahan, Delvac Oceandy, Gindo Tampubolon

**Affiliations:** ^1^Department of Public Administration, Faculty of Administrative Science, Brawijaya University, Malang, Indonesia; ^2^Department of Nursing, Faculty of Health and Education, Manchester Metropolitan University, Manchester, United Kingdom; ^3^Department of Public Health, Faculty of Medicine, Brawijaya University, Malang, Indonesia; ^4^Department of Mathematics, Physics, and Electrical Engineering, Northumbria University, Newcastle upon Tyne, United Kingdom; ^5^Division of Cardiovascular Sciences, The University of Manchester, Manchester, United Kingdom; ^6^Global Health at the Global Development Institute, The University of Manchester, Manchester, United Kingdom

**Keywords:** vaccine hesitancy, coronavirus, confidence and complacency beliefs, cross-sectional study, rural Indonesia

## Abstract

**Introduction:**

Vaccine hesitancy could undermine efforts to reduce incidence of coronavirus disease 2019 (COVID-19). Understanding COVID-19 vaccine hesitancy is crucial to tailoring strategies to increase vaccination acceptance. This study aims to investigate the prevalence of and the reasons for COVID-19 vaccine hesitancy in Malang District, Indonesia.

**Methods:**

Data come from a cross-sectional study among individuals aged 17-85 years old (N = 3,014). Multivariate ordered logistic regression was used to identify factors associated with postponing or refusing COVID-19 vaccines. The Oxford COVID-19 vaccine hesitancy scale was used to measure vaccine hesitancy. A wide range of reasons for hesitancy, including coronavirus vaccine confidence and complacency, vaccination knowledge, trust and attitude in health workers and health providers, coronavirus conspiracy, anger reaction and need for chaos, populist views, lifestyle, and religious influence, was examined.

**Results and discussion:**

The results show that 60.2% of the respondents were hesitant to receive the COVID-19 vaccine. Low confidence and complacency beliefs about the vaccine (OR = 1.229, 95% CI = 1.195–1.264) and more general sources of mistrust within the community, particularly regarding health providers (OR = 1.064, 95% CI = 1.026–1.102) and vaccine developers (OR = 1.054, 95% CI = 1.027–1.082), are associated with higher levels of COVID-19 vaccine hesitancy. Vaccine hesitancy is also associated with anger reactions (OR = 1.019, 95% CI = 0.998–1.040), need for chaos (OR = 1.044, 95% CI = 1.022–1.067), and populist views (OR = 1.028, 95% CI = 1.00–1.056). The findings were adjusted for socio-demographic factors, including age, sex, education, marital status, working status, type of family, household income, religious beliefs, and residency. The results suggest the need for an effective health promotion program to improve community knowledge of the COVID-19 vaccine, while effective strategies to tackle “infodemics” are needed to address hesitancy during a new vaccine introduction program.

## Introduction

The coronavirus disease 2019 (COVID-19) pandemic continues to evolve and impact communities around the world, including in Indonesia. Since the first case was reported in December 2019, as of April 1, 2022, the pandemic has caused more than 6 million infectious disease cases and 155,164 deaths in Indonesia. The COVID-19 vaccine is a vital pillar in recovery from the pandemic (1), yet its full potential will be realized only if we can overcome vaccine hesitancy. According to the World Health Organization (WHO), vaccine hesitancy is a delay, disapproval or refusal of vaccinations notwithstanding the availability of vaccination services (2).

Vaccine hesitancy is a crucial problem that must be addressed given the increasing frequency of vaccine concerns and the requirement to immediately maintain high vaccination coverage across the country in order to reduce the effects of the current coronavirus pandemic. Potential disease epidemics caused by vaccine hesitancy would result in unnecessary pain and death for a large portion of the population as well as a waste of limited local health department resources. The COVID-19 vaccine is considered one of the most effective ways to protect individual health, secure the most vulnerable groups, restore social and economic life, and perhaps attain population health and safety through immunity (3). However, high levels of uptake are necessary for COVID-19 vaccinations to be effective. Vaccine hesitancy may thus lead to significant risks for the hesitant individual as well as the wider community.

Vaccine hesitancy has multifactorial and complex causes that entail a variety of interventions at the individual, medical, community, and healthcare system levels (4). These multifactorial causes, however, are frequently viewed through the lens of complacency, lack of confidence, and low convenience (5). Complacency occurs when the perceived risks of vaccine-preventable diseases are low, so vaccination is not considered necessary. A person's decision to accept or reject a vaccine can be viewed as a trade-off between risk and benefit. Vaccine hesitancy occurs when the public perceive the urgency of vaccination as low (referred to as complacency) and have concerns about the efficacy and safety of the vaccination (referred to as confidence).

Confidence includes trust in a vaccine's safety and efficacy, the healthcare system that provide the services, and the motivations of policymakers who make vaccine decisions. A lack of trust in vaccination is worsened by a poor understanding of vaccine effectiveness as well as mistrust in government and healthcare authorities and in the vaccine's innovativeness. In addition, the ease of obtaining vaccination (referred to as convenience) may be considered. Vaccination convenience is important when it comes to physical availability, affordability, and accessibility. Other reasons for hesitancy that have been recognized include social processes such as norms, lack of altruistic purposes, and lack of collective responsibility (5).

The COVID-19 vaccination program in Indonesia started in January 2021. In the early days of the pandemic, the government set up two phases for delivering COVID-19 vaccination. Phase 1 (January–April 2021) focused on health workers and support staff, medical students, older people (60+ years old), and front-line workers such as public transport drivers, army and police. Phase 2 (April–March 2021) focused on individuals younger than 60 years old with comorbidities. If vaccine doses were available, additional individuals were to be vaccinated. Ten COVID-19 vaccines are allowed by the government authority, including Sinovac, AstraZeneca, Sinopharm, Moderna, Pfizer, Novavax, Sputnik-V, Janssen, Convidencia, and Zifivax. All COVID-19 vaccines are available for free in public healthcare (1). Despite the availability of COVID-19 vaccination in the country, Indonesia had a low vaccination uptake, with roughly 67% of the adult population did the COVID-19 vaccine by May 2021. Hence, this study aimed to assess and identify factors associated with COVID-19 vaccine hesitancy in Indonesia, focusing on Malang, the second-largest district in East Java Province.

## Materials and methods

### Study setting

This study was conducted in Malang District, East Java Province, Indonesia, between July 07 and August 02, 2021, when the second wave of COVID-19 reached Indonesia. The COVID-19 vaccination program had already been launched in the district. Malang is the second-largest district in East Java Province, with a population of 2,542,963 people (2015 census) distributed across 33 sub-districts and 390 villages, 273 (70%) of which are rural and 117 (30%) of which are urban. It has 39 primary health centers, or *Pusat Kesehatan Masyarakat* (*Puskesmas*; one for every 65,000 people), and 390 village health clinics, or *Pondok Kesehatan Desa* (*Ponkesdes*; one for every 7,000 people). Malang District classifies 10.15% of its population as “poor or near poor,” compared to 11.46% in East Java overall (6). The Malang authority carried out its first COVID-19 vaccination program in January 2021. The government provided 1 million doses of vaccine in the first period. It then provided an additional 2 million doses beginning in April 2021. To accelerate the rollout of vaccination, the government has been employing front-line health workers in all 390 village health clinics to deliver vaccination.

### Study design and participants

This study was a cross-sectional study among individuals aged 17–85 years. KoboToolbox (a simple, robust, and powerful data collection tool) was used to generate a semi-structured questionnaire (7). The survey apps were utilized by 39 trained field researchers in charge of data collection. The sampling population was determined using a stratified-based sampling design, with the population stratified into urban and rural areas. The total population for rural areas was 1,780,074 individuals and that for urban areas was 762,889 individuals. Based on the confidence level of 99.9% and the margin of error of 5%, we found the minimum samples for rural and urban areas to be 1,082 and 1,081 individuals, respectively. Initially, 3,600 potential participants (1,990 for rural areas and 1,610 for urban areas) provided written informed consent. To encourage participants to participate in the survey, we provided a door prize for 10 randomly selected participants at the end of the survey. Of these, 3,014 completed the survey (1,698 for rural areas and 1,316 for urban areas), yielding an 83.7 percent response rate.

### Measures

#### Vaccine hesitancy scale

The Oxford COVID-19 vaccine hesitancy scale was used to measure hesitancy. This scale consists of seven items (see online Supplementary material). Higher scores indicate higher levels of vaccine hesitancy. In addition, Shapiro et al.s' vaccine hesitancy scale was employed to test the convergent validity of the Oxford COVID-19 vaccine hesitance scale (8). The questions were translated into Bahasa Indonesia and re-translated following the Oxford COVID-19 vaccine hesitancy scale guidelines. A language expert from Brawijaya University performed the initial translation into Bahasa Indonesia. Then, two independent language experts were hired as outside translators; they translated the questions back into English. The English re-translation agreed with the original questionnaire in English. The set of translated questions was pre-tested on 42 respondents. The pre-test stratified respondents by age, gender, and education. The results showed that even those with little formal education were able to understand the questions correctly. Similar procedures of questionnaire adaption were implemented for other scales used in this study.

#### Coronavirus vaccine confidence and complacency scale

The Oxford COVID-19 vaccine confidence and complacency scale was used to assess respondents' confidence in and complacency regarding COVID-19 vaccines (9). It assesses attitudes of vaccine complacency (e.g., the collective value of vaccine and the belief that an individual could contract the coronavirus and the vaccine would not work) and confidence (e.g., regarding vaccine innovation speed and side effects). The responses to each item were coded from 1 to 5. A “don't know” option was also available, but it was not scored. Higher scores imply a greater level of negative attitudes toward the vaccine.

#### Vaccination knowledge scale

The vaccination knowledge measure developed by Zingg and Siergrist was used to measure respondents' knowledge about vaccines (10). Participants were asked to assess a set of statements as correct or incorrect. Incorrect or “do not know” answers were scored as zero, while accurate/correct answers were counted as one. As a result, higher scores imply a greater understanding of vaccines.

#### Trust in doctors and developers questionnaire

The Oxford trust in doctors and developers questionnaire was used to measure trust in doctors and vaccine developers. This questionnaire includes 11 items about interpersonal disrespect from doctors and five items about distrust in vaccine developers (9). Each item was assessed on a scale of 1 (totally disagree) to 4 (absolutely agree), with a “don't know” option that was not scored. Higher scores suggest that respondents found doctors to be more disrespectful and less respected, and that respondents had more negative perceptions of vaccine developers.

#### Attitudes toward doctors and medicine questionnaire

Nineteen items from Marteau's questionnaire on doctors and medicine were used to measure respondents' attitudes toward doctors and medicine (11). Each item was graded on a scale of 1 (strongly disagree) to 6 (strongly agree). Higher scores indicate more positive attitudes toward doctors and medicine, whereas lower scores suggest more negative attitudes toward doctors and medicine.

#### The MacArthur scale of subjective social status

The MacArthur scale of subjective social status, consisting of two different items, was used to examine where people saw themselves on a social ladder compared to other people in their social circle (12). Each item has a rating of 0–10. Higher scores indicate a poorer subjective social position.

#### Brief core schema scales

The brief core schema scale developed by Fowler et al. was used to assess respondents' beliefs about themselves (13). Twelve items examine beliefs about oneself, ranging from “do not believe” (0) to “completely believe” (4). Higher scores indicate greater agreement with the items.

#### Medical doctor practice assessment questionnaire

A general practice assessment questionnaire with eight items was used to evaluate how respondents had been treated by their doctors (14). Each item was rated from 1 (very good) to 5 (very poor). Higher scores suggest less pleasant experiences with doctors.

#### Primary care or Puskesmas experience questionnaire

This study used eight questions to assess favorable and unfavorable experiences of primary community care. Each item is scored on a three-point scale ranging from 1 (no) to 3 (yes) (9). Higher ratings imply that respondents had fewer favorable primary care experiences and more negative primary care experiences.

#### OCEANS coronavirus conspiracy scale

The OCEANS coronavirus conspiracy scale, consisting of a seven-item general conspiracy scale and a 14-item COVID-19 conspiracy scale was used to measure respondents' levels of belief in coronavirus conspiracy theories (9). Each item was assigned a value from 1 (“do not agree”) to 5 (“totally agree”). A “don't know” response option that was not factored into the score was also provided. Higher scores imply a stronger belief in coronavirus conspiracy theories.

#### Vaccine conspiracy beliefs scale

A seven-item questionnaire was employed that asked participants how strongly they agreed with vaccine conspiracy statements on a seven-point scale (8). Higher scores reflect stronger support for conspiracy theories.

#### Everyday discrimination scale

This study used William et al.s' everyday discrimination scale, which has nine items (15). On a scale of 1 (almost every day) to 6 (never), individuals were asked to rate how frequently they found themselves in nine bad situations. Higher scores suggest that respondents have had fewer discriminatory experiences.

#### Dimensions of anger reactions

Five items of anger reactions from Forbes et al., assessed on a scale of 1 (none) to 5 (all the time), were employed (16). Higher scores imply a higher level of anger.

#### Need for chaos

Eleven items to assess the “need for chaos” were used to measure respondents' desire to undermine the established political system to raise one's social position. They are rated on a scale of 1 (strongly disagree) to 7 (strongly agree) (17). Higher scores imply a greater need for chaos.

#### Lifestyle and economic/government liberty

Seven items from Iyer et al. were used to assess the libertarian worldviews of respondents (18). Responses range from 1 (strongly disagree) to 6 (strongly agree). Higher scores reflect more libertarian views.

#### Populist views

Five questions from Akkerman et al. were used to assess the populist views of respondents. Each was scored on a scale of 1 (strongly disagree) to 6 (strongly agree) (19). Higher scores imply more populist views.

#### Perceived religious influence on health behavior and illness as punishment by God for sin

The Holt et al. questionnaire was used to measure the impact of religion on an individual's health beliefs (20). Each of the 15 questions was evaluated on a scale of 1 (strongly disagree) to 4 (strongly agree). Higher scores imply that religion has a bigger influence on health behavior and that disease is viewed as a punishment.

Socio-demographic factors include age, sex (female = 1, male = 0), education (elementary or less = 0, junior secondary school = 1, high school or higher education = 2), marital status, employment status (unemployed = 1, employed = 0), job status: whether affected by the pandemic or not, place of living (urban = 1, rural = 0), family monthly income [< Indonesian rupiah (IDR) 1 million = 1, IDR 1–3 million = 2, IDR 3–5 million = 3, IDR > 5 million = 4], and religion (Muslim = 1, non-Muslim = 0) (21). The Indonesian language version of the questionnaire used in the study is available in Supplementary material 1.

### Statistical analysis

To ensure that the sample was representative of people living in Malang at large, descriptive statistics [percentages and 95% confidence intervals (CI)] for the outcomes were generated using cross-sectional weights. Since the independent variable was an ordinal scale, ordered logistic regression was performed (22). STATA 17.1 was used to clean and analyse the data. Listwise deletion was used to remove missing data from the analyses, allowing each model to include a different number of participants.

### Ethics and consent

The survey was prefaced with a participant information statement and consent form in simple Bahasa (the local language). A trained interviewer read the statement and consent for every participant *via* the KoboToolbox survey app and confirmed that participants had understood the participant information statement to proceed to the survey; completion of the survey constituted consent. Ethics approval was granted by the Brawijaya University Ethical Board (Reference: 11/EC/KEPK/04/2021).

## Results

### Sample characteristics

Table 1 describes the socio-demographic characteristics of the respondents. Overall, the characteristics of the study sample are similar to the district's socio-demographic characteristics (7). The average age of respondents was 32 years old (standard deviation or SD = 9.5), which is similar to the average age of the district population in 2021. In 2021, the proportion of the female population in Malang was 49.6%, which is comparable to the proportion of female respondents in our study (49%).

**Table 1:** Sample characteristics and bivariate correlation with COVID-19 vaccine hesitancy.

**Variables**	**Mean or %**	**SD**	**Odds ratio**	**95% CI**	
				**Lower**	**Upper**
Age	32.39	9.52	0.99	0.99	1.00
Female	49%		1.13^**^	1.00	1.29
**Education level**					
Elementary or less	2%		Ref.		
Junior secondary	16%		0.63	0.35	1.11
High school	18%		0.67	0.38	1.17
College	64%		0.62^*^	0.35	1.07
**Marital status**					
Single	25%		Ref.		
Married	72%		0.60^***^	0.52	0.69
Divorced	2%		1.11	0.71	1.74
Widowed	2%		1.45	0.86	2.44
**Working status**					
Job not affected by the pandemic	53%		Ref.		
Job affected by the pandemic	47%		0.92	0.81	1.04
Employed	90%		Ref.		
Unemployed	10%		1.46^***^	1.19	1.79
**Type of family**					
Nuclear family	61%		Ref.		
Joint family	39%		1.30^***^	1.15	1.47
**Household income**					
<1 million rupiah	57%		Ref.		
1–3 million rupiah	35%		1.69^***^	1.47	1.96
3–5 million rupiah	5%		2.00^***^	1.48	2.69
>5 million rupiah	3%		3.40^***^	2.32	5.01
**Religious belief**					
Muslim	99%		Ref.		
Non-Muslim	1%		0.46^**^	0.22	0.97
**Residency**					
Rural	78%		Ref.		
Urban	22%		1.26^**^	1.08	1.47

* Significant at 0.01.

** Significant at 0.05.

*** Significant at 0.001.

However, the proportion of respondents who graduated from college or university in this study was higher than that of the general population. Almost two-thirds (64%) of the study respondents graduated from college or university, while the proportion of individuals who graduated from college in the district in 2021 was about 36%. Most respondents in this study were married (72%) and employed (90%). These proportions are comparable with the proportion of the Malang population in 2021. Almost half of respondents (47%) reported that their job was affected by the pandemic, including losing their job or having their work hours reduced. The proportion of respondents living with their parents or other family members was 61%, and 57% reported having a monthly per capita income of <IDR 1 million (equal to USD 70). It was reported in 2021 that 10.5% of the Malang population had a daily expenditure of <USD 1.00). Most of the respondents in this study were Muslim (99%) and lived in rural areas (78%); these figures are nearly identical to the proportions of the Malang population in 2021. Results of unadjusted ordered logistic regressions show that being female, less educated, unemployed, single, living in a joint family, earning a higher income, being Muslim, and living in an urban area were associated with higher COVID-19 vaccine hesitancy.

### Vaccine hesitancy

Table 2 describes respondents' responses to each of the questions on COVID-19 vaccine hesitancy. Only 39.8% reported that they would definitely take the vaccine if the government offered it to them. About 44.6% of the respondents reported wanting to get the vaccine as soon as possible. Likewise, 42.4% of respondents reported that they would get the vaccine as soon as possible when it became available through *Puskesmas* or primary healthcare. Accordingly, 23.3% of respondents said they would get the vaccine at *Puskesmas* when they had time. Regarding attitudes toward receiving the COVID-19 vaccine, 37.3 and 23.3% of respondents were very keen and quite positive about it. However, less than half of respondents (40.9%) reported that they would strongly encourage their family or friends to get the vaccination. Less than half of respondents (39.8%) said they were eager to get the vaccine, while 28.0% were willing to. Accordingly, 12.1% of respondents reported being anti-vaccination, and 14.0% said that being vaccinated was either unimportant or very unimportant.

**Table 2:** Distribution of responses on each of the Oxford COVID-19 vaccine hesitancy items.

**If offered, would you take a COVID-19 vaccine (approved for use in Indonesia)?**	***N* (%)**
Definitely	1,199 (39.8%)
Probably	706 (23.4%)
I may or I may not	558 (18.5%)
Probably not	299 (9.9%)
Definitely not	118 (3.9%)
Don't know	134 (4.4%)
**If there is a COVID-19 vaccine available:**	
I will want to get it as soon as possible	1,344 (44.6%)
I will take it when offered	847 (28.1%)
I am not sure what I will do	364 (12.1%)
I will put off (delay) getting it	138 (4.6%)
I will refuse to get it	185 (6.1%)
Don't know	136 (4.5%)
**I would describe my attitude toward receiving a COVID-19 vaccine as:**	
Very keen	1,125 (37.3%)
Pretty positive	700 (23.2%)
Neutral	534 (17.7%)
Quite uneasy	311 (10.3%)
Against it	200 (6.6%)
Don't know	144 (4.8%)
**If a COVID-19 vaccine was available at my** ***Puskesmas*****, I would:**	
Get it as soon as possible	1,279 (42.4%)
Get it when I have time	700 (23.2%)
Delay getting it	349 (11.6%)
Avoid getting it for as long as possible	241 (8.0%)
Never get it	243 (8.1%)
Don't know	202 (6.7%)
**If my family or friends were thinking of getting a COVID-19 vaccination, I would:**	
Strongly encourage them	1,233 (40.9%)
Encourage them	596 (19.8%)
Not say anything to them about it	492 (16.3%)
Ask them to delay getting the vaccination	362 (12.0%)
Suggest that they do not get the vaccination	128 (4.2%)
Don't know	203 (6.7%)
**I would describe myself as:**	
Eager to get the COVID-19 vaccine	1,201 (39.8%)
Willing to get the COVID-19 vaccine	843 (28.0%)
Not bothered about getting the COVID-19 vaccine	416 (13.8%)
Unwilling to get the COVID-19 vaccine	188 (6.2%)
Anti-vaccination for COVID-19	366 (12.1%)
Don't know	
**Taking a COVID-19 vaccination is:**	
Really important	1,091 (36.2%)
Important	901 (29.9%)
Neither important nor unimportant	398 (13.2%)
Unimportant	264 (8.8%)
Really unimportant	157 (5.2%)
Don't know	203 (6.7%)

These questions ask how you would respond if there were an approved COVID-19 vaccine from the Indonesian government.

### Reasons for vaccine hesitancy

Table 3 describes the socio-demographic determinants of vaccine hesitancy. Being older (OR = 1.015; 95% CI = 1.005–1.025) and being female (OR = 1.360; 95% CI = 1.181–1.567) were associated with higher vaccine hesitancy. Participants with high school and college educations had less likely vaccine hesitancy than those with elementary school or less education (OR = 0.557; 95% CI = 0.290–1.069 for high school and OR = −0.432; 95% CI = 0.227–0.819 for college). Null association was found for junior secondary school. Married individuals were less likely to reject vaccination than single individuals (OR = 0.559; 95% CI = 0.442–0.708). Null associations were found for divorced and widowed individuals. Individuals who reported that their jobs had been affected by the pandemic (OR = 0.878; 95% CI = 0.761–1.014) were less likely to refuse vaccination after controlling for all covariates. There was no association between employment status or family type and vaccine hesitancy. Higher-income individuals demonstrated a higher level of vaccine hesitancy independent of all covariates. Non-Muslims were also linked with a lower level of vaccine rejection. Null association was found in the relationship between individuals living in urban areas and vaccine hesitancy. In addition, the interaction between education and household income on hesitancy was examined to influence education for household income status. We found individuals educated at the junior secondary school level or higher and from households with incomes >5 million rupiahs to be associated with less hesitancy (detailed estimate results for the interaction variables are available in Supplementary material 2).

**Table 3:** Results of multivariate ordered logistic regression measuring the association of socio-demographic variables of interest with COVID-19 vaccine hesitancy.

**Variables**	**Odds ratio**	**Std. err**.	**95% CI**	

			**Lower**	**Upper**
Age	1.015^***^	0.005	1.005	1.025
Female	1.360^***^	0.098	1.181	1.567
**Education level**				
Elementary and lower (ref.)				
Junior secondary	0.620	0.207	0.322	1.194
High school	0.557^*^	0.185	0.290	1.069
College	0.432^**^	0.141	0.227	0.819
**Marital status**				
Single (ref.)				
Married	0.559^***^	0.067	0.442	0.708
Divorced	0.798	0.206	0.481	1.324
Widowed	1.092	0.355	0.578	2.065
**Working status**				
Job not affected by the pandemic (ref.)				
Job affected by the pandemic	0.878^*^	0.064	0.761	1.014
Employed (ref.)				
Unemployed	1.208	0.192	0.886	1.649
**Type of family**				
Joint family (ref.)				
Nuclear family	1.059	0.109	0.865	1.296
Household income				
IDR < 1 million (ref.)				
IDR 1–3 million	1.904^***^	0.151	1.630	2.224
IDR 3–5 million	2.480^***^	0.386	1.828	3.366
IDR >5 million	3.687^***^	0.756	2.467	5.509
**Religious belief**				
Muslim (ref.)				
Non-Muslim	0.463^*^	0.197	0.201	1.066
**Residency**				
Rural (ref.)				
Urban	1.152	0.103	0.967	1.372

* Significant at 0.01.

** Significant at 0.05.

*** Significant at 0.001.

Table 4 shows the results from multivariate ordered logistic regressions showing the reasons for vaccine hesitancy. The regression results were adjusted with all socio-demographic factors in Table 3. Regarding confidence and complacency, lower confidence in and complacency toward the COVID-19 vaccine are associated with hesitancy (OR = 1.229, 95% CI = 1.195–1.264 for the collective importance of a COVID-19 vaccine, OR = 1.049, 95% CI = 1.011–1.089 for respondents' perceptions that they may contract the disease, OR = 1.049, 95% CI = 1.011–1.089 for the perception that vaccination is an effective solution, OR = 1.210, 95% CI = 1.165–1.258 for the rapidity with which the vaccines were developed, and OR = 1.199, 95% CI = 1.160–1.240 for vaccine side effects). However, general knowledge about vaccines (OR = 0.831, 95% CI = 0.768–0.899) and knowledge about childhood vaccines (OR = 0.952, 95% CI = 0.899–1.009) are negatively associated with hesitancy. People's trust in doctors and vaccine developers influences vaccine hesitancy. In this study, we observed that respondents' perceptions of disrespect on the part of doctors (OR = 1.055, 95% CI = 1.025–1.086) and their negative view of vaccine developers (OR = 1.055, 95% CI = 1.027–1.082) were associated with vaccine hesitancy. However, positive attitudes toward doctors, positive attitudes toward medicine, and negative attitudes toward medicine had no significant association with COVID-19 vaccine hesitancy. A negative attitude toward doctors had a positive and significant association with vaccine hesitancy (OR = 1.025, 95% CI = 0.997–1054). Negative beliefs about oneself, positive beliefs about oneself, and assessments of health workers were not associated with vaccine hesitancy. Negative experiences with public health providers were associated with higher vaccine hesitancy (OR = 1.064, 95% CI = 1.026–1.102). A higher score on the OCEANS coronavirus conspiracy scale was associated with greater vaccine hesitancy (OR = 1.011, 95% CI = 1.001–1.022), but general coronavirus conspiracy beliefs had no association with vaccine hesitancy. Other behaviors, including disrespect and having fewer discriminatory experience, were not associated with vaccine hesitancy. Anger reactions were associated with greater COVID-19 vaccine hesitancy (OR = 1.019, 95% CI = 0.998–1.040). A stronger need for chaos was also linked to COVID-19 vaccine hesitancy (OR = 1.044, 95% CI = 1.022–1.067). While lifestyle libertarians and economic/government liberty were not associated with hesitancy, populist views were significantly associated with COVID-19 vaccine hesitancy (OR = 1.028, 95% CI = 1.000–1.056). The influence of religion on health behavior was not related to COVID-19 vaccination hesitancy.

**Table 4:** Results of multivariate ordered logistic regression measuring the association of reasons for vaccine hesitancy with COVID-19 vaccine hesitancy.

**Variables**	**Odds ratio**	**Std. err**.	**95% CI**	
			**Lower**	**Upper**
Collective importance of a COVID-19 vaccine	1.229^***^	0.018	1.195	1.264
Belief that the respondent may contract COVID-19 and that the vaccine would work	1.049^**^	0.020	1.011	1.089
Speed of vaccine development	1.210^***^	0.024	1.165	1.258
Side effects	1.199^***^	0.020	1.160	1.240
General knowledge about vaccines	0.831^***^	0.033	0.768	0.899
Knowledge about childhood vaccines	0.952^*^	0.028	0.899	1.009
Interpersonal disrespect on the part of doctors	1.000	0.012	0.977	1.023
Respondent's perception of doctors' respect toward them	1.055^***^	0.015	1.025	1.086
Negative views of vaccine developers	1.054^***^	0.014	1.027	1.082
Positive attitude to doctors	1.008	0.021	0.968	1.049
Negative attitude to doctors	1.025^*^	0.015	0.997	1.054
Positive attitude to medicine	1.033	0.025	0.986	1.082
Negative attitude to medicine	1.014	0.020	0.976	1.054
Negative beliefs about self	1.004	0.011	0.982	1.026
Positive beliefs about self	0.991	0.012	0.968	1.014
Assessment of health workers	1.007	0.013	0.982	1.032
Positive experiences with public health providers	1.018	0.019	0.982	1.055
Negative experiences with public health providers	1.064^***^	0.019	1.026	1.102
OCEANS coronavirus conspiracy scale	1.011^**^	0.005	1.001	1.022
General coronavirus conspiracy beliefs	1.005	0.007	0.991	1.018
Others disrespectful	1.006	0.015	0.977	1.035
Others react negatively	1.030	0.029	0.974	1.089
Anger reactions	1.019^*^	0.011	0.998	1.040
Need for chaos	1.044^***^	0.011	1.022	1.067
Lifestyle and economic/government liberty	1.007	0.014	0.980	1.035
Populist views	1.028^**^	0.014	1.000	1.056
Religious influence on health behavior	0.979	0.015	0.949	1.010
Illness as punishment for sin	0.986	0.012	0.964	1.009

* Significant at 0.01.

** Significant at 0.05.

*** Significant at 0.001.

The results were adjusted to all socio-demographic factors in Table 3.

## Discussion

This study measured coronavirus vaccination hesitancy and its determinants in the second-largest district in East Java, Indonesia. Only 39.8% of the Malang District population was willing to get the COVID-19 vaccine. This proportion was substantially lower than those observed in prior studies in other developing countries, including China (91.3%) (23), Malaysia (94.3%) (24), Brazil (85.4%), South Africa (81.6%), Mexico (76.3%), India (74.5%), and Nigeria (65.2%) (25). A study in Indonesia in 2020 found that 93.3% of respondents wanted to be vaccinated provided that the vaccine is 95% effective and provided by the government free of cost (26). However, that study was performed before the first COVID-19 vaccine deployment in the United Kingdom on December 08, 2020. The changes in disease progression, information and social media, vaccine availability, and government policies may have affected COVID-19 vaccine hesitancy over time. A longitudinal study in the United States found a decline in pro-vaccine attitudes and in COVID-19 vaccination intentions during the 6-month study period (27). Political ideology and media exposure were among the determinants of the decline.

The high level of vaccine hesitancy in Indonesia confirms the country's low vaccine uptake. According to a Ministry of Health study brief based on data obtained between April and May 2021, vaccination uptake remained low, with roughly 67% of Indonesia's adult population likely to take the coronavirus vaccine once it became available to them. Another survey, conducted by the Center for Strategic and International Studies, noted that 63 and 55% of youth in Jakarta and Yogyakarta, respectively, did not intend to become vaccinated against COVID-19. Those two regions were the epicenter of COVID-19. Furthermore, a survey conducted by the Indonesian Medical Association between February and March 2021 reported that only 45% of Indonesians aged 22–25 intended to get a COVID-19 vaccination (28). These high proportions of hesitancy are a cause of great concern for the government, which set an optimistic target of up to 2 million doses per day to reach the national vaccination coverage target of 208 million (28).

This study found that complacency and confidence in vaccine decision-making are related to vaccine hesitancy. The findings confirm those of prior studies, which explain that a set of beliefs tightly bound to a willingness to take the COVID-19 vaccine are plausible drivers of vaccine uptake (9). Freeman et al. explained that acceptance of a vaccine is tied to beliefs about its collective importance: that a vaccine will save lives and help the community and that it will be dangerous if residents do not get vaccinated (9). This corresponds to evidence from a study on collective responsibility in the context of climate change mitigation emphasizing that collective rather than personal responsibility may lead to greater change in individual behaviors (29, 30). This study also found three other key types of beliefs about a COVID-19 vaccine to be associated with hesitancy: that a respondent thought it unlikely that they would be infected and that the vaccine would work; that the speed of development of the vaccine would affect its safety and efficacy; and that receiving the vaccine might be physically unpleasant and that the recipient would feel experimented upon. All of these findings are highly consistent with the framing in the vaccine hesitancy literature of the importance of complacency and confidence in vaccine decision-making (9).

Furthermore, prior research has identified a frequent theme of vaccination safety concerns as a factor in COVID-19 vaccine hesitancy; these safety concerns include the vaccine's potential unexplained side effects, views about the disease itself, and a general impression that vaccine trials were rushed through (29–31). These findings are also confirmed in the present study. This study found low confidence in the speed of vaccine development and concerns about side effects to be associated with vaccine hesitancy in Malang District. Earlier research highlights the impact of both factors on vaccine hesitancy. For example, prior studies have found that, rather than actual vaccine side effects, fear of side effects is one of the main reasons for which individuals refuse to be vaccinated (32). In a randomized control trial study, Sudharsanan et al. found that although COVID-19 vaccine serious side effects are rare, the media's presentation of these risks may amplify concerns. Thus, addressing public concerns over vaccine side effects will help to improve the uptake of vaccines. Likewise, prior studies have found that individuals who do not perceive COVID-19 as a deadly disease and believe that they could be easily treated may then refuse vaccination as they think that the disease does not present a danger to them (33). A review revealed that concerns about the rapid development of the COVID-19 vaccines, as well as the belief that COVID-19 vaccines are harmful and ineffective, present barriers to vaccine uptake (33). These studies also show that vaccine hesitancy is significantly associated with concerns about vaccine safety, vaccine development speed, and longer-term vaccine side effects (33), which is confirmed in the present study.

Knowledge about COVID-19 vaccines is an important determinant of vaccination acceptance. A lack of knowledge about COVID-19 vaccines creates vaccine hesitancy. In this study, the variables measuring participants' knowledge about the COVID-19 vaccines and general knowledge about the importance of childhood vaccination are both associated with lower hesitancy. These findings confirm prior studies that suggest the important roles of effective vaccine education and campaigns to address vaccine hesitancy (34, 35). Such studies suggest that individuals with knowledge and positive attitudes toward vaccines are likely to have a higher willingness to accept vaccination (36, 37). Community education regarding vaccination programs is needed to improve individual knowledge of the benefits as well as the side effects of vaccination before the inoculation campaign, especially in communities with significant exposure to misinformation about COVID-19 vaccines and vaccine fake news.

For most people, taking a vaccination is a matter of trust; they believe that the vaccine is necessary, will work as expected, and is safe (38, 39). Therefore, unwillingness to receive the vaccination is more likely when excessive mistrust is part of an individual's general attitude (40). If a person is skeptical of experts, authorities, and organizations, he or she will likely be skeptical of vaccinations. Distrust is more likely when people feel mistreated and prone to exploitation (marginalized), think that doctors look down on them, believe in conspiracy theories, embrace specific worldviews (e.g., individualism), and are ignored (e.g., exhibit a “need for chaos”) (41). A prior study in Indonesia found that trust in both science and government is linked to higher vaccine acceptance. Due to mistrust of the government among Indonesians, the country's response to the COVID-19 situation has also been delayed by denial, reluctance, and rejection (42, 43). Confirming these earlier findings, the present study also reveals that vaccine hesitation is significantly associated with certain confidence and complacency beliefs about COVID-19 vaccines and that it is correlated with sources of mistrust.

This study has found that mistrust of doctors and COVID-19 vaccine developers is related to vaccine hesitancy. These results are in line with the results of previous studies, which have shown a relationship between levels of trust in public institutions and in COVID-19 vaccination (9). However, Quinn and Fremitus reported that individuals who do not trust their government tend to refuse COVID-19 vaccination (4). In contrast to this study, the study found that only mistrust in doctors and COVID-19 vaccine developers was related to respondents' refusal of vaccines. Concerns about scientists' personal bias and corporate motivations, as well as a lack of communication with the general public about COVID-19 advances and vaccinations, are the key issues facing scientists and may result in loss of faith in them (44). This could explain two things that have been observed in Malang District and in Indonesia in general. First, even before the COVID-19 pandemic, citizens had poor perceptions of and experiences with doctors' services. Second, there is a great deal of exposure to fake news, misinformation, disinformation and infodemics about COVID-19 and COVID-19 vaccination. As has been explained in various mass media regarding conspiracy theories about COVID-19 and the COVID-19 vaccination program as well as misleading news about vaccines, people do not have faith in vaccine developers (9).

Accordingly, our findings also highlight the relationship between the OCEANS coronavirus conspiracy beliefs and vaccine hesitancy. The findings also confirm prior studies both in developed and developing countries (45, 46). Pertwee pointed out that conspiracy theories and rumors about COVID-19 and vaccines should not be understood simply as false beliefs. Rather, they can be read as expressions of popular fears and anxieties (47). A study using the conspiracy mentality and COVID-19 phobia scales found a positive correlation between the belief in conspiracy theories and increased vaccine hesitancy (48). Conspiracy theories represent attempts to impose narrative coherence on frightening pandemic situations. Many of the anxieties fuelling COVID-19 rumors and conspiracy theories long predate the pandemic; they have probably been exacerbated by the widespread social uncertainty that existed before COVID-19 pandemic. For example, issues surrounding globalization and capitalism, Muslims, and terrorism have led to anti-imperialist and anti-Western colonialist movements in some developing countries.

Anger reactions and the need for chaos are associated with hesitancy. Respondents with higher levels of anger and need for chaos are likely to reject vaccination. These findings support literature that explains the negative effect of negative emotional reactions such as anger and the need for chaos on individual vaccine decisions (16, 17). Since the beginning of the pandemic crisis, members of the public may be experiencing various emotions such as anger, fear, sadness, and anxiety. For example, some participants in the present study reported feeling angry after hearing of unexpected adverse effects or rumors about COVID-19 vaccines. These negative emotional experiences may influence participants' decisions to reject vaccination. Populist views are likewise associated with vaccine hesitancy. These findings confirm earlier studies in Europe that have found a positive association between vaccine hesitancy and political populism. These studies identify some key drivers among populists, such as distrust in institutions, elites, and experts, of refusal of vaccination programs offered by authorities (19). Some similar evidence is also revealed in the present study.

Certain socio-demographic factors show an association with vaccine hesitancy. Older people have been more hesitant to get the COVID-19 vaccine. This finding contrasts with findings in Japan (49). A review of 49 studies also revealed that youth was associated with a lower willingness to receive vaccination (33). Our finding that older people are more hesitant to get vaccinated may be due to cognitive barriers (50, 51). Older adults in Indonesia generally received fewer years of formal education and have less social contact than younger people (52). Another plausible explanation is that older adults are more susceptible to misinformation and digital exclusion (38). Among the risk factors for vaccine hesitancy in this study were being female, having a high income with little education, being Muslim, and living in an urban area. According to studies from the United States, women are more likely to believe that the COVID-19 vaccine is harmful (39). However, this result requires further investigation as a prior study found that immunization rates among women for other vaccines, including influenza, were higher than those among men (41). Our results that respondents with higher incomes exhibit greater vaccine hesitancy contrast with prior studies (37). However, this correlation was indeed found, especially for high earners with low levels of education. On the other hand, the intention to accept the vaccine was observed to differ among various socio-economic groups (50, 51). People living under different socio-economic conditions may have different views regarding COVID-19 vaccination. Religiosity was negatively correlated with COVID-19 vaccination, and we observed that some people were avoiding vaccination on religious grounds (37, 53). Our findings further emphasize the necessity of education in increasing COVID-19 vaccine acceptance. People with less education have a lower acceptance rate (37). Lower parental educational level is also a predictor of refusal of COVID-19 vaccine uptake among children (32).

### Limitations

The present study is not without limitations. First, the design of this study is cross-sectional, and the pandemic continues to evolve. Vaccine hesitancy may change due to various factors, including public health interventions, the appearance of new viral variants, and new vaccine availability. Longitudinal surveys should thus be performed to examine how vaccine hesitancy evolves. Second, in addition to quantitative surveys, a qualitative analysis could be employed to improve understanding of factors related to vaccine hesitancy. Third, invitations to participate were distributed through e-mail and text messages to the participants. The sample for this research did not include any potential responders without internet access. Further study with wider sampling should be undertaken to identify factors of vaccine hesitancy.

### Implications

Vaccine hesitancy in Indonesia as observed in this study is quite high compared to that found in other countries. One plausible explanation for this is that many vaccine-preventable infectious diseases are still causing a substantial number of deaths annually in Indonesia. The unsuccessful efforts to tackle vaccine-preventable infectious diseases in the country may lead to a lower perceived need for or value of the COVID-19 vaccine. High-income countries, in contrast, have successfully eliminated vaccine-preventable diseases; therefore, more people are confident in the impact of the COVID-19 vaccine. Our findings could contribute to overcoming misunderstandings about public health, particularly regarding vaccination. Providing accurate knowledge about COVID-19 and especially regarding vaccinations, using simple language so that people of all socio-economic and educational backgrounds can understand, may enhance health literacy and vaccine awareness. A variety of personalized, simple-to-understand health communications delivered *via* several modalities may help people make better-informed health decisions and increase their likelihood of getting the COVID-19 vaccination. Our findings show that specific populations, such as older people, who are prone to digital exclusion, have a higher level of vaccine hesitancy. Using traditional media such as television, newspaper, or radio to inform the population about COVID-19 could thus be a beneficial choice for the government.

The COVID-19 vaccine initiative is a crucial pillar in the struggle against COVID-19. Currently, governments and policymakers worldwide are racing to expand the vaccination program as they believe that the program's effectiveness is key to public health interventions fighting the virus. Effectiveness is defined here as achieving high uptake among adult inhabitants, preferably enough to produce herd immunity of the country's population. Effectiveness also entails equal access, acceptability, and delivery to prevent disparities in care and disease outcomes. Widespread acceptance of vaccines is vital to achieving sufficient immunization coverage so that the pandemic can be brought to an end. However, vaccine hesitancy could continue to undermine efforts to control the coronavirus.

## Data availability statement

The raw data supporting the conclusions of this article will be made available by the authors, without undue reservation.

## Ethics statement

The studies involving human participants were reviewed and approved by Brawijaya University Ethical Board (Reference: 11/EC/KEPK/04/2021). The participants provided their written informed consent to participate in this study. Written informed consent to participate in this study was provided by the participants' legal guardian/next of kin.

## Author contributions

S, H, RS, and SA prepared the study design. S, H, and RS collected data and conducted data analysis. S and RS wrote the main manuscript text. S, AM, SA, EP, DO, and GT reviewed the manuscript. All authors contributed to the article and approved the submitted version.
