# Supplementary material for: Understanding COVID-19 vaccine hesitancy: A cross-sectional study in Malang District, Indonesia
Source: Front Public Health. 2023 Jan 26;10:1030695. doi: 10.3389/fpubh.2022.1030695 (PMC9909106; doi:10.3389/fpubh.2022.1030695)
Supplement: Supplementary file 2 [file Data_Sheet_1.PDF]

# Survei persepsi tentang vaksin Covid-19

Kami ingin menanyakan beberapa hal tentang pendapat Anda mengenai vaksinasi Covid-19. Survei ini kami lakukan untuk memahami bagaimana persepsi masyarakat tentang vaksin Covid-19. Silakan jawab dengan jujur apa adanya. Jawaban Anda hanya akan kami gunakan untuk kepentingan riset ini.

## Pandangan tentang vaksin Covid-19

Pertanyaan-pertanyaan ini menanyakan tanggapan Anda tentang vaksin telah disetujui oleh Kementerian Kesehatan Indonesia

1. 1. Jika Anda ditawari untuk vaksinasi Covid-19, apakah Anda [...] bersedia? *

- Sangat bersedia
- Mungkin bersedia
- Ragu-ragu
- Menolak
- Sangat menolak
- Tidak tahu

2. 2. Jika ada program vaksinasi Covid-19 gratis [...] *

- Saya ingin melakukannya sesegera mungkin
- Saya akan melakukannya jika ditawari
- Saya tidak yakin apa yang akan saya lakukan
- Saya akan menunda untuk melakukannya
- Saya akan menolak untuk melakukannya
- Tidak tahu

3. 3. Sikap Anda terhadap vaksin Covid-19 [...] *

- Sangat mendukung
- Cukup  mendukung
- Netral
- Masih bingung
- Menentangnya
- Tidak tahu

4. 4. Jika Puskesmas, Rumah sakit, atau Dinkes menghubungi saya untuk vaksinasi Covid-19, Anda akan [...] *

- Sesegera mungkin mendaftar
- Mendaftar
- Menunggu hingga diingatkan kembali
- Menunggu hingga dihubungi berkali-kali
- Tidak pernah mendaftar
- Tidak tahu
- Saya tidak menggunakan handphone

5. 5. Jika vaksin Covid-19 tersedia di Puskesmas terdekat, Anda akan [...] *

- Melakukannya sesegera mungkin
- Melakukannya ketika waktu saya longgar
- Menunda untuk melakukan vaksin
- Menghindarinya sebisa mungkin
- Tidak pernah ingin di vaksin
- Tidak tahu

6. 6. Jika keluarga atau kawan saya ingin melakukan vaksin Covid-19, Anda akan [...] *

- Sangat mendukung mereka
- Mendukung mereka
- Tidak berkomentar apapun terkait hal itu
- Meminta mereka untuk menunda melakukan vaksinasi
- Menyarankan mereka untuk tidak melakukan vaksinasi
- Tidak tahu

7. 7. Andaikan Anda memiliki anak, dan sekolah anak Anda meminta izin anak Anda untuk di vaksin Covid-19, maka Anda akan [...] *

- Pasti mengizinkannya
- Agak mengizinkannya
- Mungkin mengizinkannya
- Mungkin tidak mengizinkannya
- Pasti tidak mengizinkannya
- Tidak tahu

8. 8. Jika seluruh penduduk diminta oleh pemerintah untuk mengikuti vaksinasi Covid-19, Anda akan [...] *

- Pasti mengikutinya
- Menerimanya
- Ragu dan menundanya
- Menolaknya
- Pasti menolaknya
- Tidak tahu

9. 9. Jika keluarga atau teman-teman saya menyarankan agar saya mengikuti vaksinasi covid-19, Anda akan [...] *

- Pasti menurutinya
- Agak menurutinya
- Mungkin menurutinya
- Menolak menurutinya
- Menyampaikan pada mereka bahwa mereka salah
- Tidak tahu

10. 10. Jika vaksin Covid-19 tersedia di Polindes/Ponkesdes terdekat maka Anda akan [...] *

- Memperolehnya sesegera mungkin
- Memperolehnya ketika waktu saya longgar
- Menunda untuk melakukannya
- Menghindarinya sebisa mungkin
- Tidak pernah ingin melakukan vaksin
- Tidak tahu

11. 11. Anda adalah seseorang yang [...] *

- Bersemangat untuk melakukan vaksinasi Covid-19
- Bersedia untuk melakukan vaksinasi Covid-19
- Tidak peduli terhadap vaksinasi Covid-19
- Tidak bersedia untuk melakukan vaksinasi Covid-19
- Anti terhadap vaksinasi Covid-19
- Tidak tahu

12. 12. Melakukan vaksinasi Covid-19 adalah [...] *

- Sangat penting
- Penting
- Antara penting dan tidak penting
- Tidak penting
- Sangat tidak penting
- Tidak tahu

13. 13. Andaikan Anda memiliki anak : *

- Saya pasti akan memberikan mereka vaksin Covid-19
- Saya akan berfikir untuk memberikan mereka vaksin Covid-19
- Saya ragu untuk memberikan mereka vaksin Covid-19
- Saya tidak akan memberikan mereka vaksin Covid-19
- Sangat akan memastikan bahwa mereka tidak akan mendapatkan vaksin covid-19
- Tidak tahu

14. 14. Jika dokter memberikan saran untuk mengulang vaksinasi covid-19 setiap tahun, Anda akan [...] *

- Mengikutinya setiap tahun tanpa terlewati
- Kemungkinan besar mengikutinya setiap tahun
- Mungkin mengikutinya setiap tahun
- Kemungkinan besar tidak akan mengikutinya tiap tahun
- pasti tidak mengikutinya tiap tahun
- Tidak tahu

15. 15. Anda akan melakukan vaksinasi covid-19 untuk melindungi keluarga dan teman-teman Anda: *

- Sangat setuju
- Agak setuju
- Ragu-ragu
- Agak tidak setuju
- Sangat tidak setuju
- Tidak tahu

16. 16. Apakah Anda berfikir Anda akan terinfeksi Covid-19 selama 12 bulan kedepan? *

- Pasti
- Agak
- Mungkin
- Mungkin tidak
- Pasti tidak
- Tidak tahu

17. 17. Vaksin COVID-19 kemungkinan besar akan: *

- Efektif untuk semua orang
- Efektif untuk sebagian besar orang
- Saya tidak yakin seberapa besar akan efektif
- Tidak efektif untuk sebagian besar orang
- Tidak akan efektif terhadap siapapun
- Tidak tahu

18. 18. Vaksin COVID-19 kemungkinan besar akan: *

- Sangat manjur untuk saya
- Agak manjur untuk saya
- Bisa iya bisa tidak manjur untuk saya
- Mungkin tidak manjur untuk saya
- Sangat tidak manjur untuk saya
- Tidak tahu

19. 19. Jika vaksin Covid-19 tersedia: *

- Sangat penting bagi saya untuk mendapatkannya
- Akan cukup membantu jika saya mendapatkannya
- Tidak masalah apakah saya mendapatkannya atau tidak
- Saya ragu saya membutuhkannya
- Saya tidak akan membutuhkannya
- Tidak tahu

20. 20. Jika Anda memperoleh vaksin Covid-19, maka akan [...] *

- Sangat menolong orang-orang disekitar saya
- Menolong orang-orang disekitar saya
- Tidak ada gunanya untuk orang-orang disekitar saya
- Tidak menolong orang-orang disekitar saya
- Sangat tidak menolong orang-orang disekitar saya
- Tidak tahu

21. 21. Jika warga masyarakat seperti Anda mendapatkan vaksin Covid-19, maka akan [...] *

- Menyalamatkan kehidupan banyak orang
- Menyelamatkan kehidupan beberapa orang
- Tidak berdampak apapun
- Menambah beberapa kematian
- Menambah sejumlah besar kematian
- Tidak tahu

22. 22. Akan__________jika vaksinasi Covid-19 semakin cepat dikembangkan dan diuji *

- Sangat baik
- Baik
- Tidak berdampak apapun
- Buruk
- Sangat buruk
- Tidak tahu

23. 23. Akan__________jika vaksinasi Covid-19 semakin cepat dikembangkan dan diuji *

- Sangat aman
- Aman
- Tidak berdampak apapun pada keselamatan
- Tidak aman
- Sangat tidak aman
- Tidak tahu

24. 24. Jika banyak orang tidak mendapatkan vaksinasi Covid-19, maka [...] *

- Akan berbahaya
- Mungkin berbahaya
- Tidak akan memberikan dampak apapun
- Mungkin akan menjadi baik
- Akan menjadi baik
- Tidak tahu

25. 25. [.......] jika mendapatkan vaksin Covid-19. *

- Akan sangat aman
- Akan aman
- Tidak jelas apakah akan aman atau tidak
- Akan tidak aman
- Akan sangat tidak aman
- Tidak tahu

26. 26. Menerima vaksinasi Covid-19, rasanya... *

- Hampir tidak terasa sakit
- Sedikit tidak menyenangkan
- Agak tidak menyenangkan
- Menyakitkan
- Sangat menyakitkan
- Tidak tahu

27. 27. Vaksinasi Covid-19 terhadap orang-orang akan memiliki efek samping yang [...] *

- Tidak ada
- Ringan
- Sedang
- Tinggi
- Mengancam hidupnya
- Tidak tahu

28. 28. Vaksin Covid-19 akan [...] *

- Sangat memperkuat sistem kekebalan saya
- Memperkuat sistem kekebalan saya
- Tidak akan memperkuat maupun memperlemah sistem kekebalan
- Memperlemah sistem kekebalan saya
- Sangat memperlemah sistem kekebalan saya
- Tidak tahu

29. 29. Jika vaksin Covid-19 yang ditawarkan dibuat oleh luar negeri, bukan asli dibuat di Indonesia, maka akan [...] *

- Sama-sama baiknya
- Cukup lebih buruk
- Lebih buruk
- Berpotensi membahayakan
- Membahayakan
- Tidak tahu

30. 30. Jika Anda mendapatkan Covid-19 [...] *

- Saya barangkali akan dirawat di rumah sakit
- Saya akan sakit parah
- Rasanya hanya seperti mendapatkan flu ringan
- Tidak akan terasa sakit
- Akan sangat baik-baik saja
- Tidak tahu

31. 31. Dengan melakukan vaksin Covid-19, Anda [...] *

- Terlindungi dari ancaman infeksi
- Cukup terlindungi dari ancaman infeksi
- Tidak yakin akan terlindungi dari ancaman infeksi
- Tidak terlindungi dari ancaman infeksi
- Sama sekali tidak terlindungi dari ancaman infeksi
- Tidak tahu

32. 32. Melakukan vaksin Covid-19 akan [...] *

- Memberi saya kebebasan penuh melanjutkan hidup normal seperti sebelumnya
- Memberi saya kebebasan yang lebih besar untuk melanjutkan hidup normal seperti sebelumnya
- Tidak akan berdampak pada kebebasan saya untuk melanjutkan hidup normal seperti sebelumnya
- Akan membatasi kebebasan saya
- Akan membatasi sepenuhnya kebebasan saya untuk melanjutkan kehidupan normal seperti sebelumnya
- Tidak tahu

33. 33. Dengan melakukan Vaksin Covid-19 menandakan: *

- Kekuatan seseorang yang luar biasa
- Kekuatan seseorang
- Tidak menandakan kekuatan atau kelemahan seseorang
- Kelemahan seseorang
- Kelemahan seseorang yang luar biasa
- Tidak tahu

34. 34. Dengan melakukan vaksin Covid-19 yang baru akan membuat saya terasa seperti kelinci percobaan: *

- Tidak setuju
- Sedikit setuju
- Agak setuju
- Setuju
- Sangat setuju
- Tidak tahu

35. 35. Saya berfikir bahwa dalam kelompok/lingkungan saya: *

- Setiap orang akan melakukan vaksin Covid-19
- Sebagian besar orang akan melakukan vaksin Covid-19
- Sekitar setenganya akan melakukan vaksin Covid-19
- Hanya beberapa saja yang akan melakukan vaksin Covid-19
- Tidak satupun orang akan melakukan vaksin Covid-19
- Tidak tahu

36. 36. Menerima vaksin Covid-19 akan membuat Anda merasa GELISAH: *

- Tidak benar sama sekali
- Sedikit benar
- Agak benar
- Benar
- Sangat benar
- Tidak tahu

37. 37. Menerima vaksin Covid-19 akan membuat Anda merasa SENANG: *

- Tidak benar sama sekali
- Sedikit benar
- Agak benar
- Benar
- Sangat benar
- Tidak tahu

38. 38. Menerima vaksin Covid-19 akan membuat Anda merasa LEGA: *

- Tidak benar sama sekali
- Sedikit benar
- Agak benar
- Benar
- Sangat benar
- Tidak tahu

39. 39. Menerima vaksin Covid-19 akan membuat Anda merasa MARAH: *

- Tidak benar sama sekali
- Sedikit benar
- Agak benar
- Benar
- Sangat benar
- Tidak tahu

40. 40. Menerima vaksin Covid-19 akan membuat Anda merasa JIJIK: *

- Tidak benar sama sekali
- Sedikit benar
- Agak benar
- Benar
- Sangat benar
- Tidak tahu

41. 41. Menerima vaksin Covid-19 akan membuat Anda merasa BAHAGIA: *

- Tidak benar sama sekali
- Sedikit benar
- Agak benar
- Benar
- Sangat benar
- Tidak tahu

42. 42. Menerima vaksin Covid-19 akan membuat Anda merasa TERHINA: *

- Tidak benar sama sekali
- Sedikit benar
- Agak benar
- Benar
- Sangat benar
- Tidak tahu

Pandangan atas Vaksin Anak-anak

Berikut kami tanyakan pendapat Anda tentang vaksinasi secara umum pada anak-anak (balita). Vaksinasi yang dimaksud BUKAN khusus tentang vaksin Covid-19. Untuk tiap-tiap pertanyaan, tentukan sejauh mana Anda setuju atau tidak setuju.

43. 1. Vaksin anak sangat penting untuk kesehatan anak *

- Sangat tidak setuju
- Tidak Setuju
- Netral
- Setuju
- Sangat setuju

44. 2. Vaksin anak sangat efektif mencegah penyakit *

- Sangat tidak setuju
- Tidak Setuju
- Netral
- Setuju
- Sangat setuju

45. 3. Memiliki anak yang sudah divaksin sangat penting untuk kesehatan orang lain disekitar kita *

- Sangat tidak setuju
- Tidak Setuju
- Netral
- Setuju
- Sangat setuju

46. 4. Semua vaksin anak yang diberikan pemerintah sangat bermanfaat *

- Sangat tidak setuju
- Tidak Setuju
- Netral
- Setuju
- Sangat setuju

47. 5. Vaksin-vaksin baru membawa lebih banyak risiko daripada vaksin lama *

- Sangat tidak setuju
- Tidak Setuju
- Netral
- Setuju
- Sangat setuju

48. 6. Informasi yang saya terima tentang vaksin dari pemerintah dapat diandalkan dan dapat dipercaya *

- Sangat tidak setuju
- Tidak Setuju
- Netral
- Setuju
- Sangat setuju

49. 7. Vaksinasi adalah cara yang baik untuk melindungan anak-anak dari penyakit *

- Sangat tidak setuju
- Tidak Setuju
- Netral
- Setuju
- Sangat setuju

50. 8. Orang-orang harus melakukan apa yang dokter sarankan untuk memvaksin anak *

- Sangat tidak setuju
- Tidak Setuju
- Netral
- Setuju
- Sangat setuju

51. 9. Saya khawatir tentang efek negatif yang ditimbulkan dari vaksin *

- Sangat tidak setuju
- Tidak Setuju
- Netral
- Setuju
- Sangat setuju

**Pengetahuan tentang Vaksinasi secara umum**

Berikut ini pendapat Anda tentang vaksinasi secara umum. Bukan spesifik tentang vaksin Covid-19. Untuk masing-masing pernyataan, harap pilih jawaban yang menurut Anda benar, salah atau tidak tahu.

52. 1. Vaksin itu tidak perlu karena semua penyakit dapat diobati tanpa harus melalui vaksin *

- Benar
- Salah
- Tidak tahu

53. 2. Sudah ada vaksin pun, penyakit cacar tetap akan ada. *

- Benar
- Salah
- Tidak tahu

54. 3. Kemanjuran vaksin mencegah penyakit telah terbukti *

- Benar
- Salah
- Tidak tahu

55. 4. Anak-anak akan lebih kebal jika mereka tidak divaksin *

- Benar
- Salah
- Tidak tahu

56. 5. Vaksin bisa menyebakan penyakit-penyakit seperti autism, gangguan saraf (pada otak, mata dan tulang belakang), serta diabetes. *

- Benar
- Salah
- Tidak tahu

57. 6. Sistem kekebalan anak tidak akan terganggu karena memperoleh banyak vaksinasi. *

- Benar
- Salah
- Tidak tahu

58. 7. Pemberian vaksin pada anak menyebabkan sistem kekebalan tubuh anak tidak * berkembang.

- Benar
- Salah
- Tidak tahu

59. 8. Dosis vaksin yang diberikan dokter TIDAK berbahaya untuk anak *

- Benar
- Salah
- Tidak tahu

60. 9. Vaksinasi menyebabkan terjadinya alergi. *

- Benar
- Salah
- Tidak tahu

## Pengalaman terkait COVID-19

Kami menanyakan pengalaman Anda tentang Covid-19

61. 1. Pernahkah Anda terinfeksi Covid-19? *

- Ya, Saya pernah mendapatkan hasil tes yang positif
- Tidak, Saya mendapatkan hasil tes negatif
- Saya pikir pernah tapi saya belum pernah tes
- Saya tidak pernah terinfeksi tapi saya belum dites
- Lainnya

62. 2. Apakah Anda termasuk kelompok berisiko jika terinfeksi Covid-19 (misalnya di atas 70 tahun, hamil, memiliki komorbid seperti hipertensi, diabetes, asma)? *

- Risiko rendah
- Risiko sedang
- Risiko tinggi

## Pertanyaan tentang protokol Covid-19

Sejauh mana yang Anda lakukan untuk mengikuti protokol kesehatan terkait Covid-19

63. 1. Jika bertemu dengan orang-orang (baik di dalam rumah maupun diluar) selain keluarga serumah, saya akan membatasi paling banyak 6 orang dan selalu menjaga jarak *

- Tidak sama sekali
- Kadang-kadang
- Beberapa kali
- Seringkali
- Selalu

64. 2. Saya berkumpul diluar rumah dengan selain keluarga serumah, namun tetap membatasi maksimal 6 orang dan menerapkan jaga jarak dengan mereka *

- Tidak sama sekali
- Kadang-kadang
- Beberapa kali
- Seringkali
- Selalu

65. 3. Membatasi jumlah orang yang Anda temui, terutama dalam waktu yang singkat *

- Tidak sama sekali
- Kadang-kadang
- Beberapa kali
- Seringkali
- Selalu

66. 4. Tidak mengadakan atau menghadiri pertemuan yang cukup sulit untuk menerapkan jaga jarak dan menghindari kontak langsung. *

- Tidak sama sekali
- Kadang-kadang
- Beberapa kali
- Seringkali
- Selalu

67. 5. Hanya bermalam di luar rumah dengan sejumlah orang tidak lebih dari 6 orang *

- Tidak sama sekali
- Kadang-kadang
- Beberapa kali
- Seringkali
- Selalu

68. 6. Anda bersedia memberikan kontak Anda kepada petugas agar Anda dapat dihubungi ketika diperlukan tes Covid-19. *

- Tidak sama sekali
- Kadang-kadang
- Beberapa kali
- Seringkali
- Selalu

69. 7. Selalu menggunakan masker didalam ruangan gedung. *

- Tidak sama sekali
- Kadang-kadang
- Beberapa kali
- Seringkali
- Selalu

70. 8. Mencuci tangan dengan sabun dan air sesering mungkin, selama paling tidak 20 detik *

- Tidak sama sekali
- Kadang-kadang
- Beberapa kali
- Seringkali
- Selalu

71. 9. Saat Anda pergi keluar, tetap jaga jarak 2 meter dari orang-orang lain setiap waktu *

- Tidak sama sekali
- Kadang-kadang
- Beberapa kali
- Seringkali
- Selalu

## Protokol kesehatan Covid-19

Kami menanyakan tentang sejauhmana Anda menaati protokol kesehatan Covid-19 sebagaimana ditetapkan pemerintah.

72. 1. Secara umum, seberapa sering Anda menaati prokes pemerintah terkait pencegahan Covid-19? *

- Tidak taat sama sekali
- Kadang-kadang
- Beberapa kali taat
- Seringkali taat
- Selalu taat

73. 2. Seberapa patuh Anda pada peraturan pemerintah terkait pencegahan Covid-19 di masa yang akan datang? *

- Tidak sama sekali
- Kadang-kadang
- Beberapa kali
- Seringkali
- Selalu

| | |
|---|---|
| Mohon sebut yang akan ada lakukan | Silakan sebut apa yang Anda akan lakukan |

74. 1. Melakukan tes Covid-19 jika ditawari ? (Untuk mengetahui apakah Anda terinfeksi) *

- Sangat setuju
- Agak setuju
- Mungkin setuju
- Mungkin tidak setuju
- Sangat tidak setuju
- Tidak tahu

75. 2. Melakukan tes antibodi Covid-19 jika ditawari (untuk memeriksa jika sebelumnya Anda terinfeksi COvid-19) *

- Sangat setuju
- Agak setuju
- Mungkin setuju
- Mungkin tidak setuju
- Sangat tidak setuju
- Tidak tahu

Pertanyaan tentang Dokter dan Pencipta Vaksin

Untuk tiap-tiap pernyataan, silahkan tentukan sejauhmana Anda setuju atau tidak setuju

Dokter....

Tentang dokter

76. 1. Saya percaya pada para dokter *

- Sangat tidak setuju
- Tidak setuju
- Setuju
- Sangat Setuju
- Tidak tahu

77. 2. Para dokter tidak peduli terhadap saya *

- Sangat tidak setuju
- Tidak setuju
- Setuju
- Sangat Setuju
- Tidak tahu

78. 3. Para dokter mengutamakan kebutuhan terbaik saya *

- Sangat tidak setuju
- Tidak setuju
- Setuju
- Sangat Setuju
- Tidak tahu

79. 4. Para dokter merendahkan saya *

- Sangat tidak setuju
- Tidak setuju
- Setuju
- Sangat Setuju
- Tidak tahu

80. 5. Para dokter tidak terlalu menghormati saya *

- Sangat tidak setuju
- Tidak setuju
- Setuju
- Sangat Setuju
- Tidak tahu

81. 6. Para dokter berusaha melakukan yang terbaik *

- Sangat tidak setuju
- Tidak setuju
- Setuju
- Sangat Setuju
- Tidak tahu

82. 7. Para dokter tidak tahu seperti apa hidup saya *

- Sangat tidak setuju
- Tidak setuju
- Setuju
- Sangat Setuju
- Tidak tahu

83. 8. Para dokter seringkali membuat kesalahan *

- Sangat tidak setuju
- Tidak setuju
- Setuju
- Sangat Setuju
- Tidak tahu

84. 9. Kepentingan para dokter hanya untuk meraup uang *

- Sangat tidak setuju
- Tidak setuju
- Setuju
- Sangat Setuju
- Tidak tahu

85. 10. Para dokter akan tetap memberikan saya vaksin walaupun hal tersebut buruk untuk saya *

- Sangat tidak setuju
- Tidak setuju
- Setuju
- Sangat Setuju
- Tidak tahu

86. 11. Saya tidak percaya dengan para dokter *

- Sangat tidak setuju
- Tidak setuju
- Setuju
- Sangat Setuju
- Tidak tahu

Penemu Vaksin Covid-19 adalah orang yang [...]

Pendapat Anda pada penemu/pengembang vaksin Covid-19

87. 12. Mengutamakan keamanan *

- Sangat tidak setuju
- Tidak Setuju
- Setuju
- Sangat Setuju
- Tidak tahu

88. 13. Tidak saya percayai *

- Sangat tidak setuju
- Tidak Setuju
- Setuju
- Sangat Setuju
- Tidak tahu

89. 14. Hanya memikirkan uang *

- Sangat tidak setuju
- Tidak Setuju
- Setuju
- Sangat Setuju
- Tidak tahu

90. 15. Tidak peduli terhadap kepentingan menolong orang lain *

- Sangat tidak setuju
- Tidak Setuju
- Setuju
- Sangat Setuju
- Tidak tahu

91. 16. Mereka tidak memeriksa dengan benar terkait keamanan vaksin Covid-19 *

- Sangat tidak setuju
- Tidak Setuju
- Setuju
- Sangat Setuju
- Tidak tahu

## Sikap terhadap Dokter dan Obat

Untuk tiap pernyataan, pilihlah apakah Anda setuju apa tidak.

Petunjuk Menjawab:
1. Sangat tidak setuju
2. Tidak setuju
3. Cenderung tidak setuju
4. Cenderung setuju
5. Setuju
6. Sangat setuju

92. 1. Semua dokter adalah dokter yang baik *

| Sangat tidak setuju |
| --- |
| 1 |
| 2 |
| 3 |
| 4 |
| 5 |
| Sangat setuju |

93. 2. Saya percaya penuh pada semua dokter di puskesmas/rumah sakit *

Sangat tidak setuju

1 ☐

2 ☐

3 ☐

4 ☐

5 ☐

Sangat setuju

94. 3. Tidak peduli walaupun jika pergi ke dokter harus antri lama, hasilnya sangat penting untuk kesembuhan penyakit saya *

| Sangat tidak setuju |
| --- |
| 1 |
| 2 |
| 3 |
| 4 |
| 5 |
| Sangat setuju |

95. 4. Para dokter mengetahui apa yang terbaik untuk Saya *

| | Sangat tidak setuju |
|---|---|
| 1 | |
| 2 | |
| 3 | |
| 4 | |
| 5 | |
| | Sangat setuju |

96. 5. Saya hanya berkonsultasi dengan dokter jika sakit saya sudah kritis *

| Sangat tidak setuju |
|---|
| 1 |
| 2 |
| 3 |
| 4 |
| 5 |
| Sangat setuju |

97. 6. Para dokter selalu menyalahkan pasien mereka jika pengobatannya tidak berhasil menyembuhkan penyakit pasien *

| | |
|---|---|
| | Sangat tidak setuju |
| 1 | |
| 2 | |
| 3 | |
| 4 | |
| 5 | |
| | Sangat setuju |

98. 7. Tidak akan ada kesepakatan antar dua dokter terkait penyakit seseorang *

| Sangat tidak setuju |
| --- |
| 1 |
| 2 |
| 3 |
| 4 |
| 5 |
| Sangat setuju |

99. 8. Para dokter sering buru buru meresepkan obat penenang kepada pasiennya *

Sangat tidak setuju

1

2

3

4

5

Sangat setuju

100. 9. Peran dokter sangat penting untuk menjaga kita tetap sehat *

| | Sangat tidak setuju |
|---|---|
| 1 | |
| 2 | |
| 3 | |
| 4 | |
| 5 | |
| | Sangat setuju |

101. 10. Saya tidak suka dokter dan orang-orang medis *

| | |
|---|---|
| | Sangat tidak setuju |
| 1 | |
| 2 | |
| 3 | |
| 4 | |
| 5 | |
| | Sangat setuju |

102. 11. Ilmu kedokteran didasarkan pada prinsip ilmiah *

.

| | Sangat tidak setuju |
| --- | --- |
| 1 | |
| 2 | |
| 3 | |
| 4 | |
| 5 | |
| | Sangat setuju |

103. 12. Kesehatan penduduk Indonesia disebabkan oleh pengobatan yang efektif *

Sangat tidak setuju

1 ○

2 ○

3 ○

4 ○

5 ○

Sangat setuju

104. 13. Pengobatan medis selama ini mampu menyembuhkan sebagian besar penyakit *

Sangat tidak setuju

1

2

3

4

5

Sangat setuju

105. 14. Dokter adalah profesi terbaik yang dimiliki oleh seseorang *

| Sangat tidak setuju |
|---|
| 1 |
| 2 |
| 3 |
| 4 |
| 5 |
| Sangat setuju |

106. 15. Pekerjaan dokter adalah mulya tetapi juga menjadi jahat ketika dokter menyelewengkannya untuk kepentingan pribadinya *

| Sangat tidak setuju |
|---|
| 1 |
| 2 |
| 3 |
| 4 |
| 5 |
| Sangat setuju |

107. 16. Nasihat dari para dokter sebagian besar adalah hanya omong kosong *

Sangat tidak setuju

1 ☐

2 ☐

3 ☐

4 ☐

5 ☐

Sangat setuju

108. 16. Nasihat dari para dokter sebagian besar adalah berdasarkan akal sehat *

| Sangat tidak setuju |
| --- |
| 1 |
| 2 |
| 3 |
| 4 |
| 5 |
| Sangat setuju |

109. 17. Banyak obat-obatan yang hanya berupa obat yang hanya menghilangkan rasa sakit sementara saja dan tidak menyembuhkan penyakit yang diderita *

Sangat tidak setuju

1

2

3

4

5

Sangat setuju

110. 18. Seringkali satu-satunya tujuan dari pemeriksaan yang dilakukan oleh dokter adalah hanya untuk menyenangkan pasien saja *

Sangat tidak setuju

1 ○
2 ○
3 ○
4 ○
5 ○

Sangat setuju

111. 19. Kebanyakan pemeriksaan yang dilakukan dokter hanya untuk rutinitas daripada untuk tujuan memeriksa penyakit tertentu *

| | Sangat tidak setuju |
| --- | --- |
| 1 | |
| 2 | |
| 3 | |
| 4 | |
| 5 | |
| | Sangat setuju |

## Pandangan tentang diri sendiri

Silakan jawab sesuai kondisi Anda

112. 1. Bayangkan tangga di bawah ini sebagai gambaran tingkat ekonomi masyarakat Indonesia *

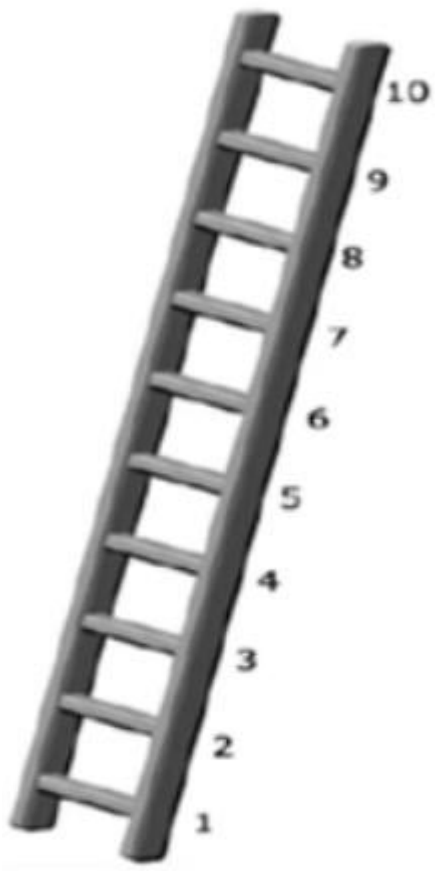


Puncak tangga (10) menggambarkan orang-orang terbaik dalam segala hal (uang paling banyak, paling intelektual, dan profesi paling dihormati).

Tangga terendah (1) menunjukkan orang – orang dengan kondisi terburuk (paling sedikit keuangannya, tingkat pendidikan rendah dan memiliki profesi paling tidak dihormati/pengangguran)
Jika posisi Anda pada tangga yang lebih tinggi berarti Anda lebih dekat dengan orang-orang terbaik, dan jika posisi lebih rendah berarti Anda lebih dekat dengan orang-orang kelas bawah.

Pada tangga nomor berapa posisi Anda berada? [angka...]

1
2
3
4
5
6
7
8
9
10

113. 2. Setiap orang memiliki kedudukan yang berbeda-beda di lingkungan/kelompoknya. Bayangkan tangga di bawah ini sebagai gambaran status sosial masyarakat Indonesia. *

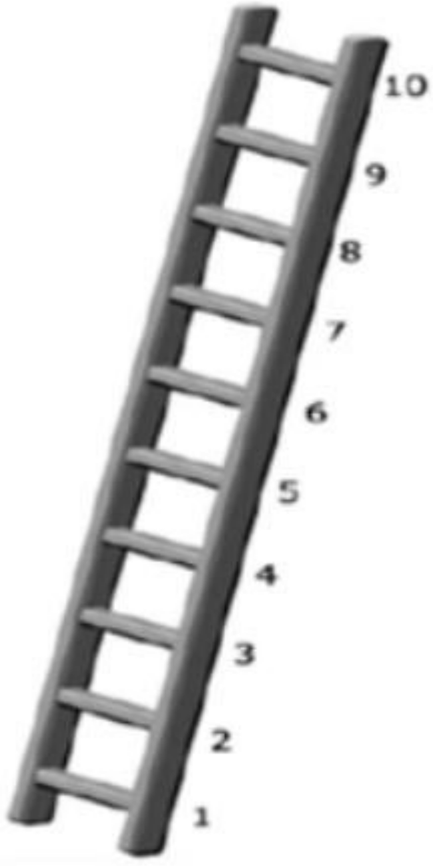


Puncak tangga (10) menggambarkan orang-orang yang memiliki kedudukan tertinggi di lingkungannya

Tangga terendah (1) menunjukkan orang – orang yang memiliki kedudukan paling rendah di lingkungannya
Silahkan pilih tangga yang paling sesuai yang menggambarkan kedudukan Anda.
Pada tangga nomor berapa posisi Anda berada? [angka...]

1

2

3

4

5

6

7

8

9

10

Keyakinan tentang diri sendiri

Seksi ini berisi tentang pandangan dan penilaian atas diri sendiri. Harap tunjukkan bagaimana Anda mempercayai setiap kalimat dengan memilih 0-4

114. Coba nilai diri Anda, seberapa percaya Anda dalam memandang diri sendiri selama seminggu terakhir. *

| | Tidak percaya | Sedikit percaya | Agak percaya | Percaya | Sangat percaya |
|---|---|---|---|---|---|
| Saya tidak dicintai | ○ | ○ | ○ | ○ | ○ |
| Saya tidak berharga | ○ | ○ | ○ | ○ | ○ |
| Saya lemah | ○ | ○ | ○ | ○ | ○ |
| Saya rapuh | ○ | ○ | ○ | ○ | ○ |
| Saya adalah orang yang buruk | ○ | ○ | ○ | ○ | ○ |
| Saya adalah seseorang yang gagal | ○ | ○ | ○ | ○ | ○ |
| Saya dihormati | ○ | ○ | ○ | ○ | ○ |
| Saya berharga | ○ | ○ | ○ | ○ | ○ |
| Saya berbakat | ○ | ○ | ○ | ○ | ○ |
| Saya adalah orang yang sukses | ○ | ○ | ○ | ○ | ○ |
| Saya adalah orang yang baik | ○ | ○ | ○ | ○ | ○ |
| Saya menarik | ○ | ○ | ○ | ○ | ○ |

## Pengalaman tentang Pelayanan Kesehatan

Kami tertarik dengan pengalaman tertentu Anda dengan dokter Anda. Seberapa Baik praktik Dokter dalam hal berikut ini:

115. 1. Membuat Anda merasa nyaman? *

- Sangat baik
- Baik
- Memuaskan
- Buruk
- Sangat Buruk
- Tidak dilakukan

116. 2. Bersikap sopan dan perhatian? *

- Sangat baik
- Baik
- Memuaskan
- Buruk
- Sangat Buruk
- Tidak dilakukan

117. 3. Mendengarkan Anda *

- Sangat baik
- Baik
- Memuaskan
- Buruk
- Sangat Buruk
- Tidak dilakukan

118. 4. Menyediakan waktu yang cukup untuk Anda *

- Sangat baik
- Baik
- Memuaskan
- Buruk
- Sangat Buruk
- Tidak dilakukan

119. 5. Memeriksa kondisi kesehatan Anda? *

- Sangat baik
- Baik
- Memuaskan
- Buruk
- Sangat Buruk
- Tidak dilakukan

120. 6. Menjelaskan kondisi kesehatan Anda dan pengobatannya? *

- Sangat baik
- Baik
- Memuaskan
- Buruk
- Sangat Buruk
- Tidak dilakukan

121. 7. Melibatkan Anda dalam pengambilan keputusan terkait perawatan Anda? *

- Sangat baik
- Baik
- Memuaskan
- Buruk
- Sangat Buruk
- Tidak dilakukan

122. 8. Menyediakan dan menyusun perawatan untuk Anda? *

- Sangat baik
- Baik
- Memuaskan
- Buruk
- Sangat Buruk
- Tidak dilakukan

## Tentang dokter dan petugas kesehatan

Tanggapan Anda tentang dokter dan petugas kesehatan

123. Seberapa besar kemungkinan Anda menyarankan praktik dokter / penyedia layanan kesehatan Anda kepada teman dan keluarga jika mereka membutuhkan perawatan atau perawatan serupa? *

- Sangat mungkin menyarankan
- Mungkin menyarankan
- Netral
- Cenderung tidak mungkin
- Sangat tidak mungkin menyarankan
- Tidak tahu

Kami ingin menanyakan pengalaman Anda memperoleh layanan di Puskesmas/Penyedia Faskes, maupun BPJS

124. Sudahkah berbagai hal berikut ini terjadi? *

| | Tidak | Mungkin | Ya | Belum dilakukan |
|---|---|---|---|---|
| Menerima pelayanan yang sangat baik | | | | |
| Diberikan antrian untuk pelayanan | | | | |
| Para karyawan telah berusaha keras untuk melayani | | | | |
| Menerima perawatan berbahaya | | | | |
| Keluarga menerima perawatan yang berbahaya | | | | |

125. Apakah Anda berfikir [...] *

| | Tidak | Mungkin | Ya | Belum dilakukan |
|---|---|---|---|---|
| Lingkungan/Kelompok Anda di jaga baik oleh pelayanan kesehatan (Poskesdes/puskesmas) | | | | |
| Masalah kesehatan lingkungan/kelompok Anda diabaikan oleh poskesdes/puskesmas | | | | |
| Pelayanan kesehatan Anda memperlakukan Anda dengan buruk | | | | |

## Pemahaman umum tentang virus COVID-19

Untuk setiap pernyataan, silahkan pilih sejauh mana Anda setuju atau tidak setuju

126. 1. Virus Covid-19 itu hoax *

- Tidak setuju
- Sedikit setuju
- Agak setuju
- Cukup setuju
- Sangat setuju
- Tidak tahu

127. 2. Virus Covid-19 adalah rekayasa manusia *

- Tidak setuju
- Sedikit setuju
- Agak setuju
- Cukup setuju
- Sangat setuju
- Tidak tahu

128. 3. Penyebaran virus Covid-19 adalah upaya yang disengaja untuk mengurangi jumlah penduduk bumi. *

- Tidak setuju
- Sedikit setuju
- Agak setuju
- Cukup setuju
- Sangat setuju
- Tidak tahu

129. 4. Penyebaran virus Covid-19 adalah upaya yang disengaja oleh pemerintah untuk tetap berkuasa *

- Tidak setuju
- Sedikit setuju
- Agak setuju
- Cukup setuju
- Sangat setuju
- Tidak tahu

130. 5. Penyebaran virus Covid-19 adalah upaya yang sengaja dibuat sekelompok orang untuk menghasilkan uang dari penjualan vaksin, obat dan alat kesehatan. *

- Tidak setuju
- Sedikit setuju
- Agak setuju
- Cukup setuju
- Sangat setuju
- Tidak tahu

131. 6. Penyebaran virus adalah upaya yang disengaja oleh satu negara untuk membuat negara Indonesia hancur *

- Tidak setuju
- Sedikit setuju
- Agak setuju
- Cukup setuju
- Sangat setuju
- Tidak tahu

132. 7. Penyebaran virus merupakan upaya yang disengaja oleh para pengusaha obat dan vaksin untuk mengendalikan dunia *

- Tidak setuju
- Sedikit setuju
- Agak setuju
- Cukup setuju
- Sangat setuju
- Tidak tahu

Pemahaman Khusus tentang virus Covid-19

133. 8. Covid-19 adalah senjata biologis yang dikembangkan oleh China dan Amerika dan sekutunya untuk menghancurkan Indonesia *

- Tidak setuju
- Sedikit setuju
- Agak setuju
- Setuju
- Sangat setuju
- Tidak tahu

134. 9. Covid-19 adalah senjata biologis yang diciptakan oleh Amerika Serikat *

- Tidak setuju
- Sedikit setuju
- Agak setuju
- Setuju
- Sangat setuju
- Tidak tahu

135. 10. PBB dan WHO telah menciptakan Covid-19 untuk mengendalikan dunia *

- Tidak setuju
- Sedikit setuju
- Agak setuju
- Setuju
- Sangat setuju
- Tidak tahu

136. 11. Yahudi telah menciptakan virus Covid untuk meruntuhkan ekonomi agar mereka berkuasa. *

- Tidak setuju
- Sedikit setuju
- Agak setuju
- Setuju
- Sangat setuju
- Tidak tahu

137. 12. Kaum kafir telah menciptakan virus Covid-19 untuk mengendalikan dunia. *

- Tidak setuju
- Sedikit setuju
- Agak setuju
- Setuju
- Sangat setuju
- Tidak tahu

138. 13. Bill Gates telah menciptakan virus Covid-19 untuk mengurangi penduduk dunia. *

- Tidak setuju
- Sedikit setuju
- Agak setuju
- Setuju
- Sangat setuju
- Tidak tahu

139. 14. Big Pharma (Perusahaan farmasi raksasa dunia) telah menciptakan virus Covid-19 untuk mencari keuntungan dari vaksin *

- Tidak setuju
- Sedikit setuju
- Agak setuju
- Setuju
- Sangat setuju
- Tidak tahu

140. 1. Covid-19 sedang dimanfaatkan pemerintah yang berkuasa untuk mengontrol warga Indonesia *

- Tidak setuju
- Sedikit setuju
- Agak setuju
- Setuju
- Sangat setuju
- Tidak tahu

141. 2. COVID-19 disebabkan oleh aplikasi Android 5G yang menyebabkan radiasi melalui gelombang radio. *

- Tidak setuju
- Sedikit setuju
- Agak setuju
- Setuju
- Sangat setuju
- Tidak tahu

142. 3. Virus Covid-19 hanyalah bagian dari konspirasi kaum kafir dunia *

- Tidak setuju
- Sedikit setuju
- Agak setuju
- Setuju
- Sangat setuju
- Tidak tahu

143. 4. COVID-19 diciptakan untuk memaksa setiap orang Indonesia melakukan vaksinasi. *

- Tidak setuju
- Sedikit setuju
- Agak setuju
- Setuju
- Sangat setuju
- Tidak tahu

144. 5. Vaksin Covid-19 akan digunakan oleh kaum kafir untuk menyebarkan penyakit. *

- Tidak setuju
- Sedikit setuju
- Agak setuju
- Setuju
- Sangat setuju
- Tidak tahu

145. 6. Organisasi Kesehatan Dunia (WHO) sudah memiliki vaksin dan menyimpannya. *

- Tidak setuju
- Sedikit setuju
- Agak setuju
- Setuju
- Sangat setuju
- Tidak tahu

146. 7. Tes PCR atau antibodi adalah rencana jahat kaum kafir untuk mengumpulkan data DNA orang Indonesia. *

- Tidak setuju
- Sedikit setuju
- Agak setuju
- Setuju
- Sangat setuju
- Tidak tahu

Pernyataan tentang dunia

Silahkan nilai masing-masing pernyataan berdasarkan skala berikut:
0% : Pasti tidak benar
10% : Tidak mungkin sama sekali
20% : Sangat tidak mungkin
30% : Tidak mungkin
40% : Agak tidak mungkin
50% : Tidak memutuskan
60% : Agak mungkin
70% : Mungkin
80% : Sangat mungkin
90% : Sangat sangat mungkin
100% : Pasti

Saya berfikir bahwa.....

147. 1. ..... Banyak sesuatu yang sangat penting terjadi di dunia, dan warga Indonesia * tidak pernah diberi tahu sebenarnya hal itu.

| |
|---|
| Pasti tidak benar |

1
2
3
4
5
6
7
8
9
10

| |
|---|
| Pasti benar |

148. 2....... Politisi biasanya tidak menceritakan kepada kita tentang motif sesungguhnya dari keputusan mereka *

| Pasti tidak benar |
|---|

1 ⬭

2 ⬭

3 ⬭

4 ⬭

5 ⬭

6 ⬭

7 ⬭

8 ⬭

9 ⬭

10 ⬭

| Pasti benar |
|---|

149. 3....... Mata-mata pemerintah mengawasi secara dekat seluruh warganya khususnya lawan politiknya *

Pasti tidak benar

1 ○
2 ○
3 ○
4 ○
5 ○
6 ○
7 ○
8 ○
9 ○
10 ○

Pasti benar

150. 4. ..... Peristiwa yang terjadi di Indonesia sering kali merupakan hasil dari kegiatan agen rahasia pemerintah. *

Pasti tidak benar

1 ◯
2 ◯
3 ◯
4 ◯
5 ◯
6 ◯
7 ◯
8 ◯
9 ◯
10 ◯

Pasti benar

151. 5....... Terdapat organisasi rahasia yang secara kuat mempengaruhi pengambilan * keputusan politik Indonesia.

| Pasti tidak benar |
| --- |

1 ◯

2 ◯

3 ◯

4 ◯

5 ◯

6 ◯

7 ◯

8 ◯

9 ◯

10 ◯

| Pasti benar |
| --- |

Kepercayaan tentang Vaksinasi

Pertanyaan ini terkait Vaksin secara umum, tidak mengacu kepada vaksin COVID-19. Untuk masing-masing pernyataan pilihlah jawaban yang sesuai dengan pendapat Anda.

Petunjuk menjawab:
1 = Sangat tidak setuju
2 = Tidak setuju
3 = Cenderung tidak setuju
4 = Netral
5 = Cenderung setuju
6 = Setuju
7 = Sangat setuju

152. 1. Data mengenai keamanan vaksin telah dimanipulasi *

Sangat tidak setuju

1 ⬭
2 ⬭
3 ⬭
4 ⬭
5 ⬭
6 ⬭
7 ⬭

Sangat setuju

153. 2. Imunisasi anak itu berbahaya dan fakta tersebut ditutup-tutupi oleh pemerintah dan dokter *

| | |
|---|---|
| | Sangat tidak setuju |
| 1 | ☐ |
| 2 | ☐ |
| 3 | ☐ |
| 4 | ☐ |
| 5 | ☐ |
| 6 | ☐ |
| 7 | ☐ |
| | Sangat setuju |

154. 3. Perusahaan farmasi telah menyembunyikan bahaya vaksin agar vaksin mereka laku terjual. *

| | |
|---|---|
| | Sangat tidak setuju |
| 1 | ⬭ |
| 2 | ⬭ |
| 3 | ⬭ |
| 4 | ⬭ |
| 5 | ⬭ |
| 6 | ⬭ |
| 7 | ⬭ |
| | Sangat setuju |

155. 4. Orang-orang selama ini ditipu oleh kemanjuran vaksin *

| |
|---|
| Sangat tidak setuju |
| 1 |
| 2 |
| 3 |
| 4 |
| 5 |
| 6 |
| 7 |
| Sangat setuju |

156. 5. Data efektivitas Vaksin selama ini dimanipulasi. *

| | Sangat tidak setuju |
| --- | --- |
| 1 | ⬭ |
| 2 | ⬭ |
| 3 | ⬭ |
| 4 | ⬭ |
| 5 | ⬭ |
| 6 | ⬭ |
| 7 | ⬭ |
| | Sangat setuju |

157. 6. Orang-orang selama ini ditipu terkait keamanan penggunaan vaksin *

| Sangat tidak setuju |
| --- |
| 1 |
| 2 |
| 3 |
| 4 |
| 5 |
| 6 |
| 7 |
| Sangat setuju |

158. 7. Pemerintah mencoba menyembunyikan bahwa vaksin menyebabkan anak menjadi autis. *

| Sangat tidak setuju |
| --- |
| 1 |
| 2 |
| 3 |
| 4 |
| 5 |
| 6 |
| 7 |
| Sangat setuju |

## Pengalaman diperlakukan tidak adil dalam kehidupan sehari-hari

Dalam keseharian Anda, Berapa sering aktivitas berikut ini terjadi pada Anda?

159. 1. Anda diperlakukan secara kurang sopan oleh orang lain. *

- Hampir setiap hari
- Minimal 1 minggu sekali
- Beberapa kali dalam 1 bulan
- Beberapa kali dalam 1 tahun
- Kurang dari 1 tahun sekali
- Tidak pernah

160. 2. Anda diperlakukan secara kurang terhormat oleh orang lain. *

- Hampir setiap hari
- Minimal 1 minggu sekali
- Beberapa kali dalam 1 bulan
- Beberapa kali dalam 1 tahun
- Kurang dari 1 tahun sekali
- Tidak pernah

161. 3. Anda menerima pelayanan buruk dibandingkan orang lain saat ada di warung kopi/restoran/toko *

- Hampir setiap hari
- Minimal 1 minggu sekali
- Beberapa kali dalam 1 bulan
- Beberapa kali dalam 1 tahun
- Kurang dari 1 tahun sekali
- Tidak pernah

162. 4. Orang-orang bertindak seakan-akan Anda orang bodoh *

- Hampir setiap hari
- Minimal 1 minggu sekali
- Beberapa kali dalam 1 bulan
- Beberapa kali dalam 1 tahun
- Kurang dari 1 tahun sekali
- Tidak pernah

163. 5. Orang-orang memperlakukan Anda sebagai sosok yang menakutkan. *

- Hampir setiap hari
- Minimal 1 minggu sekali
- Beberapa kali dalam 1 bulan
- Beberapa kali dalam 1 tahun
- Kurang dari 1 tahun sekali
- Tidak pernah

164. 6. Orang-orang menganggap Anda adalah sosok yang tidak jujur *

- Hampir setiap hari
- Minimal 1 minggu sekali
- Beberapa kali dalam 1 bulan
- Beberapa kali dalam 1 tahun
- Kurang dari 1 tahun sekali
- Tidak pernah

165. 7. Anda dipanggil secara tidak sopan atau dihina *

- Hampir setiap hari
- Minimal 1 minggu sekali
- Beberapa kali dalam 1 bulan
- Beberapa kali dalam 1 tahun
- Kurang dari 1 tahun sekali
- Tidak pernah

166. 8. Anda diancam atau dilecehkan *

- Hampir setiap hari
- Minimal 1 minggu sekali
- Beberapa kali dalam 1 bulan
- Beberapa kali dalam 1 tahun
- Kurang dari 1 tahun sekali
- Tidak pernah

## Pertanyaan tentang Amarah

Pikirkan selama 1 bulan terakhir, pilih angka yang meneskpriskan berapa kali Anda merasakan perasaan tersebut.

167. 1. Saya marah pada orang-orang atau situasi tertentu *

- Tidak pernah
- Jarang
- Kadang-kadang
- Seringkali
- Hampir sepanjang hari

168. 2. Saat saya marah, saya akan semakin menjadi-jadi *

- Tidak pernah
- Jarang
- Kadang-kadang
- Seringkali
- Hampir sepanjang hari

169. 3. Saat saya marah, akan bertahan lama *

- Tidak pernah
- Jarang
- Kadang-kadang
- Seringkali
- Hampir sepanjang hari

170. 4. Ketika saya marah pada seseorang, saya ingin memukul mereka. *

- Tidak pernah
- Jarang
- Kadang-kadang
- Seringkali
- Hampir sepanjang hari

171. 5. Kemarahan saya menghalangi saya untuk bergaul dengan orang-orang seperti yang saya harapkan. *

- Tidak pernah
- Jarang
- Kadang-kadang
- Seringkali
- Hampir sepanjang hari

## Pandangan dunia

Petunjuk menjawab:
1 = Sangat tidak setuju
2 = Tidak setuju
3 = Cenderung tidak setuju
4 = Netral
5 = Cenderung setuju
6 = Setuju
7 = Sangat setuju

172. 1. Saya terkejut ketika bencana alam terjadi dimana-mana di Indonesia *

| |
|---|
| Sangat tidak setuju |
| 1 |
| 2 |
| 3 |
| 4 |
| 5 |
| 6 |
| 7 |
| Sangat setuju |

173. 2. Lembaga sosial dan masyarakat Indonesia telah mengalami pembusukan sehingga tidak berfungsi sebagaimana mestinya *

| |
|---|
| Sangat tidak setuju |
| 1 |
| 2 |
| 3 |
| 4 |
| 5 |
| 6 |
| 7 |
| Sangat setuju |

174. 3. Saya membayangkan terjadinya bencana alam yang memusnahkan sebagian besar umat manusia sehingga sekelompok kecil orang dapat memulai lagi dari awal. *

| | Sangat tidak setuju |
|---|---|
| 1 | |
| 2 | |
| 3 | |
| 4 | |
| 5 | |
| 6 | |
| 7 | |
| | Sangat setuju |

175. 4. Saya pikir masyarakat harus dimusnahkan habis *

| Sangat tidak setuju |
| --- |
| 1 |
| 2 |
| 3 |
| 4 |
| 5 |
| 6 |
| 7 |
| Sangat setuju |

176. 5. Ketika saya berpikir tentang partai politik dan para politisi, saya berfikir 'biarkan saja mereka mati semuanya' *

| |
|---|
| Sangat tidak setuju |
| 1 |
| 2 |
| 3 |
| 4 |
| 5 |
| 6 |
| 7 |
| Sangat setuju |

177. 6. Saya pikir kita tidak mampu memperbaiki masalah partai politik dan lembaga * sosial sehingga kita perlu menghancurkannya dan memulai kembali

Sangat tidak setuju

1 ⬭

2 ⬭

3 ⬭

4 ⬭

5 ⬭

6 ⬭

7 ⬭

Sangat setuju

178. 7. Kita perlu menghancurkan lembaga politik yang ada saat ini dan memulai lagi dari awal *

Sangat tidak setuju

1

2

3

4

5

6

7

Sangat setuju

179. 8. Saya bersiap-siap setiap saat ketika militer, polisi, dan negara tidak dapat lagi * melindungi saya

| Sangat tidak setuju |
| --- |
| 1 ◯ |
| 2 ◯ |
| 3 ◯ |
| 4 ◯ |
| 5 ◯ |
| 6 ◯ |
| 7 ◯ |
| Sangat setuju |

180. 9. Saya perlu kekacauan di sekitar saya *

| Sangat tidak setuju | |
|---|---|
| 1 | |
| 2 | |
| 3 | |
| 4 | |
| 5 | |
| 6 | |
| 7 | |
| Sangat setuju | |

181. 10. Terkadang saya merasa ingin menghancurkan hal-hal indah *

| |
|---|
| Sangat tidak setuju |
| 1 |
| 2 |
| 3 |
| 4 |
| 5 |
| 6 |
| 7 |
| Sangat setuju |

182. 11. Tidak ada benar dan salah di dunia ini *

| | |
|---|---|
| | Sangat tidak setuju |
| 1 | |
| 2 | |
| 3 | |
| 4 | |
| 5 | |
| 6 | |
| 7 | |
| | Sangat setuju |

Pandangan Sosial

Petunjuk menjawab:
1 = Sangat tidak setuju
2 = Tidak setuju
3 = Cenderung tidak setuju
4 = Netral
5 = Cenderung setuju
6 = Setuju
7 = Sangat setuju

183. 1. Orang yang sukses dalam bisnis berhak untuk menikmati kekayaan mereka sesuai keinginan mereka walaupun itu merugikan orang lain. *

| Sangat tidak setuju |
| --- |
| 1 |
| 2 |
| 3 |
| 4 |
| 5 |
| 6 |
| 7 |
| Sangat setuju |

184. 2. Masyarakat akan menjadi ideal jika setiap warga dibiarkan untuk bertanggung jawab atas kehidupan mereka sendiri tanpa campur tangan pemerintah *

Sangat tidak setuju

1

2

3

4

5

6

7

Sangat setuju

185. 3. Pemerintah terlalu berlebihan dalam mengatur kehidupan warga sehari-hari *

Sangat tidak setuju

1 ⬭

2 ⬭

3 ⬭

4 ⬭

5 ⬭

6 ⬭

7 ⬭

Sangat setuju

186. 4. Pemerintah Indonesia harus berbuat lebih banyak untuk memajukan kebaikan * bersama, bahkan jika perlu mengatur kebebasan individu.

| | Sangat tidak setuju |
|---|---|
| 1 | ◯ |
| 2 | ◯ |
| 3 | ◯ |
| 4 | ◯ |
| 5 | ◯ |
| 6 | ◯ |
| 7 | ◯ |
| | Sangat setuju |

187. 5. Tuan tanah bebas untuk membangun gedung semau mereka selama mereka tidak membahayakan tetangga. *

| Sangat tidak setuju |
|---|
| 1 |
| 2 |
| 3 |
| 4 |
| 5 |
| 6 |
| 7 |
| Sangat setuju |

188. 6. Saya pikir setiap orang harus diberikan kebebasan, sepanjang mereka mereka * tidak melanggar kesamaan hak orang lain.

| | |
|---|---|
| | Sangat tidak setuju |
| 1 | ◯ |
| 2 | ◯ |
| 3 | ◯ |
| 4 | ◯ |
| 5 | ◯ |
| 6 | ◯ |
| 7 | ◯ |
| | Sangat setuju |

189. 7. Setiap orang seharusnya bebas memutuskan tentang norma atau nilai kelompok/tradisi yang ingin mereka ikuti *

| | |
|---|---|
| | Sangat tidak setuju |
| 1 | ⬭ |
| 2 | ⬭ |
| 3 | ⬭ |
| 4 | ⬭ |
| 5 | ⬭ |
| 6 | ⬭ |
| 7 | ⬭ |
| | Sangat setuju |

190. 8. Politisi di Indonesia harusnya mengikuti kemauan masyarakat *

Sangat tidak setuju

1 ◯

2 ◯

3 ◯

4 ◯

5 ◯

6 ◯

7 ◯

Sangat setuju

191. 9. Rakyat yang seharusnya membuat keputusan kebijakan paling penting, bukan politisi *

| Sangat tidak setuju |
| --- |
| 1 |
| 2 |
| 3 |
| 4 |
| 5 |
| 6 |
| 7 |
| Sangat setuju |

192. 10. Saya lebih baik diwaliki oleh seorang warga yang bertanggungjawab daripada oleh seorang politisi parpol tertentu. *

| Sangat tidak setuju |
| --- |
| 1 ◯ |
| 2 ◯ |
| 3 ◯ |
| 4 ◯ |
| 5 ◯ |
| 6 ◯ |
| 7 ◯ |
| Sangat setuju |

193. 11. Pejabat yang terpilih, terlalu banyak omong dan sedikit aksi *

| Sangat tidak setuju |
| --- |
| 1 |
| 2 |
| 3 |
| 4 |
| 5 |
| 6 |
| 7 |
| Sangat setuju |

194. 12. Apa yang orang sebut "kompromi" dalam politik sebenarnya hanya dilakukan untuk mendukung kepentingan seorang pejabat. *

Sangat tidak setuju

1 ⬭
2 ⬭
3 ⬭
4 ⬭
5 ⬭
6 ⬭
7 ⬭

Sangat setuju

## Skala Otoritarian

195. 1. Saya sangat senang melihat banyak anak muda saat ini yang menentang pemerintah yang berkuasa. *

| Sangat tidak setuju |
| --- |
| 1 |
| 2 |
| 3 |
| 4 |
| 5 |
| 6 |
| 7 |
| 8 |
| 9 |
| Sangat setuju |

196. 2. Yang paling dibutuhkan oleh negara Indonesia adalah disiplin, di mana setiap warganya mengikuti pemimpinnya dalam satu kesatuan *

| | |
|---|---|
| | Sangat tidak setuju |
| 1 | |
| 2 | |
| 3 | |
| 4 | |
| 5 | |
| 6 | |
| 7 | |
| 8 | |
| 9 | |
| | Sangat setuju |

197. 3. Aturan agama terkait aborsi, pornografi, dan pernikahan harus diberlakukan dengan ketat sebelum terlambat. *

Sangat tidak setuju

1

2

3

4

5

6

7

8

9

Sangat setuju

198. 4. Tidak ada yang salah dengan hubungan seksual pranikah *

| |
|---|
| Sangat tidak setuju |
| 1 |
| 2 |
| 3 |
| 4 |
| 5 |
| 6 |
| 7 |
| 8 |
| 9 |
| Sangat setuju |

199. 5. Masyarakat Indonesia TIDAK membutuhkan pemerintahan dan regulasi yang kuat. *

| Sangat tidak setuju |
| --- |
| 1 ◯ |
| 2 ◯ |
| 3 ◯ |
| 4 ◯ |
| 5 ◯ |
| 6 ◯ |
| 7 ◯ |
| 8 ◯ |
| 9 ◯ |
| Sangat setuju |

200. 6. Fakta tentang kejahatan dan kerusuhan pada masyarakat baru-baru ini menunjukkan bahwa kita harus lebih keras menindak para pembuat onar, jika kita ingin menegakkan hukum dan ketertiban. *

| | |
|---|---|
| | Sangat tidak setuju |
| 1 | ◯ |
| 2 | ◯ |
| 3 | ◯ |
| 4 | ◯ |
| 5 | ◯ |
| 6 | ◯ |
| 7 | ◯ |
| 8 | ◯ |
| 9 | ◯ |
| | Sangat setuju |

Kepercayaan tentang agama dan kesehatan

Petunjuk Menjawab:
1= Sangat tidak setuju
2= Tidak setuju
3= Setuju
4= Sangat setuju

201. 1. Saya cenderung menghindari sesuatu yang membahayakan tubuh saya karena kepercayaan agama/spiritual saya *

Sangat tidak setuju

1 ⬭

2 ⬭

3 ⬭

4 ⬭

Sangat setuju

202. 2. Kepercayaan agama/spiritual saya memiliki pengaruh yang besar terhadap kesehatan saya *

| | |
|---|---|
| | Sangat tidak setuju |
| 1 | |
| 2 | |
| 3 | |
| 4 | |
| | Sangat setuju |

203. 3. Allah membantu saya untuk menjaga gaya hidup yang sehat *

| | |
|---|---|
| | Sangat tidak setuju |
| 1 | |
| 2 | |
| 3 | |
| 4 | |
| | Sangat setuju |

204. 4. Allah membantu saya untuk menghindari kebiasaan buruk yang menurunkan kesehatan saya *

| | |
|---|---|
| | Sangat tidak setuju |
| 1 | ◯ |
| 2 | ◯ |
| 3 | ◯ |
| 4 | ◯ |
| | Sangat setuju |

205. 5. Karena kepercayaan agama/spiritual saya, Saya tidak mengkonsumsi obat-obatan yang membahayakan tubuh saya *

| | |
|---|---|
| | Sangat tidak setuju |
| 1 | ◯ |
| 2 | ◯ |
| 3 | ◯ |
| 4 | ◯ |
| | Sangat setuju |

206. 6. Saya berusaha mempraktekkan "kehidupan yang sehat" karena ajaran agama saya *

| Sangat tidak setuju |
| --- |
| 1 |
| 2 |
| 3 |
| 4 |
| Sangat setuju |

207. 7. Karena kepercayaan agama saya, Saya tidak mengkonsumsi rokok/tembakau *

| Sangat tidak setuju |
| --- |
| 1 |
| 2 |
| 3 |
| 4 |
| Sangat setuju |

208. 8. Penyakit adalah akibat dari pikiran negatif seseorang *

Sangat tidak setuju

1 ○

2 ○

3 ○

4 ○

Sangat setuju

209. 9. Allah memberi kita penyakit untuk memperingatkan kita *

Sangat tidak setuju

1 ○

2 ○

3 ○

4 ○

Sangat setuju

210. 10. Penyakit adalah akibat dari sesuatu buruk yang dilakukan seseorang dalam hidupnya *

| Sangat tidak setuju |
| --- |
| 1 |
| 2 |
| 3 |
| 4 |
| Sangat setuju |

211. 11. Allah menjadikan penyakit untuk menghukum manusia *

| Sangat tidak setuju |
| --- |
| 1 |
| 2 |
| 3 |
| 4 |
| Sangat setuju |

212. 12. Allah menjadikan penyakit untuk menghukum manusia dari kesalahan yang telah dilakukan dalam hidupnya *

| |
|---|
| Sangat tidak setuju |
| 1 |
| 2 |
| 3 |
| 4 |
| Sangat setuju |

213. 13. Penyakit adalah hukuman Allah atas dosa atau gaya hidup manusia *

| |
|---|
| Sangat tidak setuju |
| 1 |
| 2 |
| 3 |
| 4 |
| Sangat setuju |

214. 14. Manusia terkena penyakit disebabkan karena gemar melakukan dosa *

| Sangat tidak setuju |
| --- |
| 1 |
| 2 |
| 3 |
| 4 |
| Sangat setuju |

215. 15. Allah menggunakan penyakit untuk menghukum manusia dari dosanya *

| Sangat tidak setuju |
| --- |
| 1 |
| 2 |
| 3 |
| 4 |
| Sangat setuju |

## Tentang Anda

216. 1. Usia *

_______________________________

217. 2. Jenis Kelamin *

- Laki-laki
- Perempuan

218. 3. Pendidikan terakhir Anda: *

- S3
- S2
- S1
- Diploma
- SMA
- SMP
- SD
- Tidak sekolah

219. 4. Seberapa penting agama dalam kehidupan Anda? *

| Tidak penting sama sekali |
| --- |
| 1 ☐ |
| 2 ☐ |
| 3 ☐ |
| 4 ☐ |
| 5 ☐ |
| 6 ☐ |
| 7 ☐ |
| Sangat penting |

220. 5. Status menikah *

- ☐ Single/Belum menikah
- ☐ Sudah menikah
- ☐ Cerai
- ☐ Janda pasangan meninggal

221. 6. Pekerjaan *

- Pelajar/mahasiswa
- Buruh pabrik/bangunan
- Pegawai negeri/pemerintah
- Karyawan swasta (bank, supermarket, restoran, hotel, dll)
- Petani
- Pedagang
- Ibu rumah tangga
- Part-time/paruh waktu
- Pengangguran
- Wiraswasta
- Pensiunan
- Sedang mencari pekerjaan
- Lainnya

222. 7. Apakah pekerjaan Anda berubah karena Covid-19? *

- Tidak
- Tidak, tapi saya ditempatkan di lokasi yang berbeda (cth: WFH/Bekerja dari rumah)
- Ya, jam kerja saya berkurang
- Ya, jam kerja saya meningkat
- Ya, saya sekarang cuti
- Ya, saya sekarang menganggur
- Ya, saya sekarang berganti pekerjaan (full-time)
- Ya, saya sekarang berganti pekerjaan (paruh waktu)

223. 8. Tempat tinggal *

- Tinggal bersama orangtua dan saudara
- Tinggal bersama istri/suami dan anak saja

224. 9. Pendapatan per bulan *

- < 1 juta
- 1-3 juta
- 3-5 juta
- > 5 juta
- Belum bekerja

225. 10. Agama *

- Islam
- Kristen
- Katolik
- Hindu
- Budha
- Konghucu
- Lainnya

226. 11. Tempat tinggal

- Kabupaten Malang
- Kota Malang
- Lainnya

227. 12. Pulau tempat tinggal *

- Sumatra
- Jawa
- Bali
- Kalimantan
- Sulawesi
- Nusa Tenggara
- Maluku/Papua

228. Nama pewawancara

__________________________________

Trimakasih

Terimakasih banyak atas bantuannya
